# mRNAs encoding self-DNA reactive cGAS enhance the immunogenicity of lipid nanoparticle vaccines

**DOI:** 10.1128/mbio.02506-23

**Published:** 2023-11-08

**Authors:** Dania Zhivaki, Emily A. Gosselin, Debrup Sengupta, Holly Concepcion, Chisom Arinze, Jonathan Chow, Anastasia Nikiforov, Veronica Komoroski, Carolyn MacFarlane, Caitlin Sullivan, Jonathan C. Kagan

**Affiliations:** 1 Corner Therapeutics, Watertown, Massachusetts, USA; National Institute of Allergy and Infectious Diseases, Bethesda, Maryland, USA

**Keywords:** vaccines, cGAS, innate immunity, mRNA, catalytic adjuvant, adjuvants

## Abstract

Lipid nanoparticle (LNP)-encapsulated mRNAs have emerged as effective vaccination tools to stimulate immunity. The most common application of this technology is to deliver mRNAs that encode antigenic proteins to dendritic cells (DCs), which then stimulate antigen-specific lymphocyte responses. It is unclear whether other immunostimulatory DC activities necessary for vaccine efficacy, beyond antigen presentation, can be induced via mRNA-encoded proteins. Herein, we report an mRNA encoding a self-DNA reactive variant of the enzyme cyclic GMP-AMP synthase (cGAS), known as cGAS∆N. cGAS∆N produces the cyclic dinucleotide cGAMP upon binding intra-mitochondrial DNA. cGAMP binds the protein STING, which activates innate immune responses that stimulate T cells. We found that when delivered to DCs via LNPs, mRNA-encoded cGAS∆N induced the upregulation of chemokine receptors, T cell costimulatory molecules, major histocompatibility complex proteins, pro-inflammatory cytokines and type I interferons from murine and human DCs. These activities exceeded the immunostimulatory activities of mRNA-encoded antigens delivered via LNPs. Co-immunization of mice with antigen-LNPs and cGAS∆N-LNPs led to the robust production of antigen-specific IFNγ-producing T cells. These T cell responses were durable and circulated through the lymphatics, blood, and lungs. Immunizations with antigen-LNPs alone, akin to what are used in the clinic, stimulated weak and transient T cell responses. Antibody responses to antigen-LNPs were biased towards type I isotypes when co-injected with cGAS∆N-LNPs, as compared to immunizations with antigen-LNPs alone. These findings establish the enzyme cGAS∆N as a catalytic adjuvant, which may prove useful in enhancing the immunogenicity of nucleic acid-based vaccines.

**IMPORTANCE:**

Nucleic acid-based vaccines hold promise in preventing infections and treating cancer. The most common use of this technology is to encode antigenic proteins on mRNAs that are delivered to cells via lipid nanoparticle (LNP) formulations. In this study, we discovered that immunostimulatory proteins can also be encoded on mRNAs in LNPs. We found that an active mutant of the enzyme cGAS, referred to as cGAS∆N, acts as a catalytic adjuvant in LNP-encapsulated mRNA vaccines. The delivery of cGAS∆N mRNA via LNPs in combination with antigen mRNA-LNPs led to durable antigen-specific IFNγ-producing T cells that exceeded the efficiency of antigen-LNPs similar to those currently used in the clinic. This strategy did not compromise B cell responses; rather it induced Th1-biased antibody isotypes. This work unveils new vaccine design strategies using mRNA-encoded catalytic adjuvants that could be ideal for generating CD8^+^ T cell and B cell responses for immunotherapies.

## INTRODUCTION

The process of vaccination requires the delivery of an antigen to dendritic cells (DCs), which are endowed with several activities needed for T and B cell-mediated immunity (1). Common vaccination approaches involve injecting a subject with an inactivated pathogen or its component antigenic molecules, or involve the production of recombinant antigens that are injected into the body to be captured by DCs (2). An alternative approach to antigen delivery was recently described, whereby nucleic acids encoding antigens are delivered to DCs through encapsulation into lipid nanoparticles (LNPs) or viral vectors (3, 4). In this latter scenario, antigens are produced within the vaccinated individuals, thereby bypassing the need for protein purification prior to vaccination. The speed of their production and their efficacy in preventing serious COVID-19 infections have increased interest in the use of nucleic acids as antigen-delivery vehicles (5). However, antigen presentation is but one DC activity needed to stimulate robust and durable T and B cell-mediated immunity. DCs need to be activated in a way that upregulates several additional activities that tailor adaptive immune responses against the threat encountered (6). These activities include (i) the upregulation of costimulatory molecules such as cluster of differentiation (CD) 40 and CD80, which ensure robust DC interactions with T cells in the lymph node; (ii) the secretion of polarization cytokines that differentiate naïve T cells into effector cell subsets; (iii) the production of memory signals that permit the differentiation and reactivation of previously primed T cells (7–10); and (iv) an enhancement of DC migratory activities from the site of immunization to the draining lymph node (dLN) (11). The absence of any of these activities mitigates the effectiveness of adaptive immunity. In the context of CD8^+^ T cells, a relevant memory signal produced by DCs is the family of type I interferons (IFNs) (12). The ideal vaccine would therefore activate all of these immunostimulatory properties on DCs.

The superfamily of innate immune pattern recognition receptors (PRRs) recognizes pathogen-associated molecular patterns (PAMPs) or self-encoded damage-associated molecular patterns (DAMPs). Upon detection of these innate immune agonists, PRRs stimulate several of the aforementioned DC activities needed for durable adaptive immunity (6, 13). While traditional vaccine formulations use exogenous PAMPs or DAMPs as adjuvants, it is unclear how to use adjuvants in LNP-vaccines. A commonly discussed means to this end is to take advantage of the fact that *in vitro* transcribed mRNA is recognized by the host as a PAMP, with the potential to stimulate several Toll-like Receptors (TLRs), RIG-I-like receptors (RLRs), and Protein Kinase R (PKR) (14). In this scenario, LNP vaccines use mRNAs as nucleic acid PAMPs and as a means to produce antigens. However, the potential benefit of utilizing the intrinsic immunostimulatory activity of *in vitro* transcribed mRNAs in vaccination is undermined by their toxicity and poor ability to promote antigen production (15).

The antigen production problem associated with mRNA-LNP vaccines can be circumvented by base modifications via the methylation of cytosine, adenine, and uridine in the RNA, which reduce innate immune recognition (16). These base modifications are used in the mRNA-LNP vaccines that protect against COVID-19, which are effective antigen production agents (15, 16); yet, they display weak adjuvant activity. A challenge therefore remains—how can one increase immunogenicity of LNP vaccines that contain modified mRNAs?

An alternative approach to solve the adjuvant deficiencies of mRNA-LNP vaccines would be to maintain the use of base modified mRNAs, but encode on these mRNAs proteins that serve as adjuvants. Recently, we reported a self-DNA reactive variant of the PRR cyclic GMP-AMP synthase (cGAS), called cGAS∆N (17). cGAS∆N is an enzyme that produces the cyclic dinucleotide 2′3′ cGAMP upon binding intra-mitochondrial DNA (18, 19). 2′3′ cGAMP then stimulates the protein STING, which activates the kinase TBK1, that, in turn, phosphorylates the transcription factor IRF3 (20, 21). Dimerization and nuclear translocation of phosphorylated IRF3 culminates in host defensive inflammatory responses typified by type I IFN production and IFN-stimulated gene (ISG) expression (22). cGAS-STING signaling also induces the activation of T cell costimulatory molecules, chemokines, and cytokines that enhance protective immunity. In contrast to cGAS∆N, wild-type cGAS is not present in mitochondria and is subject to several levels of regulation to prevent self-DNA reactivity (18, 23). The robust inflammatory activities associated with self-DNA reactivity by cGAS∆N suggested the utility of this protein as an enzyme-adjuvant, which we refer to herein as a catalytic adjuvant.

In this study, we found that cGAS∆N mRNA, delivered to DCs via LNPs, induced the expression of migratory chemokine receptors, T cell costimulatory molecules, major histocompatibility complex (MHC) proteins, and several cytokines, including type I IFN. These activities exceeded the immunostimulatory activities of mRNAs that encode antigens, delivered via LNPs. Consequently, co-immunization of mice with antigen-LNPs in combination with cGAS∆N-LNPs led to robust production of antigen-specific IFNγ-producing T cells. These T cell responses were durable and circulated through the lymphatics, blood, and lungs. Immunizations with antigen-LNPs alone, akin to what is used in the clinic, stimulated weak and transient T cell responses. Antibody responses to antigen-LNPs were biased toward type I isotypes when co-injected with cGAS∆N-LNPs, as compared to immunizations with antigen-LNPs alone. These findings validate the idea that cGAS∆N represents a catalytic adjuvant, which may prove useful in enhancing the immunogenicity of nucleic acid-based vaccines.

## RESULTS

### LNPs exhibit similar sizing and loading profiles, regardless of mRNA cargo sequence

**Fig 1:**
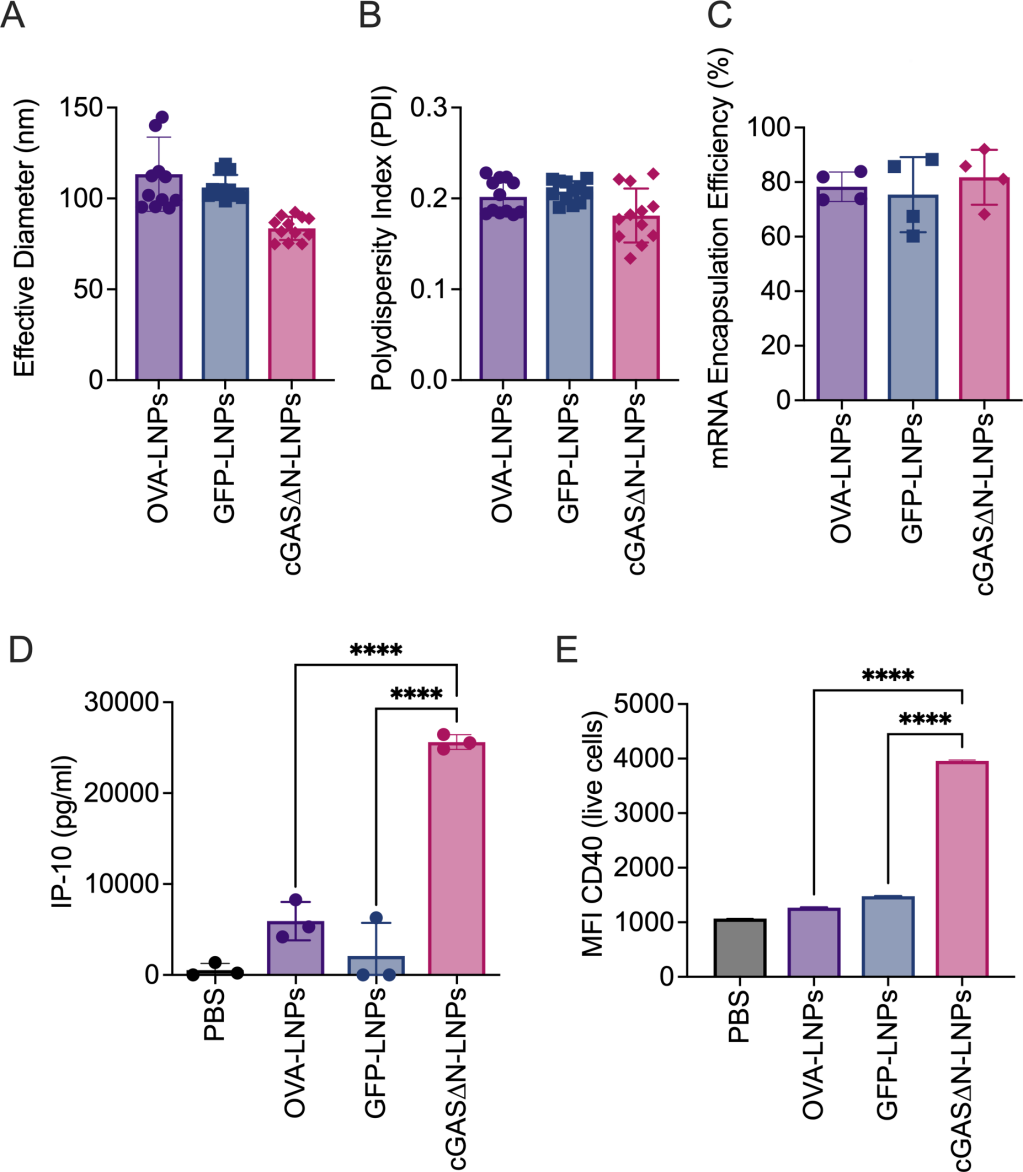
LNP characterization and THP1 activation. LNPs were prepared using OVA mRNA, GFP mRNA, and cGAS∆N mRNA. All LNPs prepared were similarly sized, with an effective diameter of around 100 nm (**A**) and relatively uniform with a polydispersity index of around 0.2 (**B**). (**C**) All mRNAs load to the LNPs at similar rates. cGAS∆N-LNPs activate THP1-Null2 cells when delivered at 1 µg/mL mRNA. (**D**) IP-10 secretion by THP1 cells was measured after 24 hours in culture with cGAS∆N-LNPs, OVA-LNPs, or GFP-LNPs. Error bars represent the standard deviation between triplicates. (**E**) CD40 median fluorescence intensity among live THP1 cells was assessed by flow cytometry 24 hours after treatment with LNPs. Error bars represent the standard error of the mean (SEM) in fluorescence intensity of cells expressing CD40. Comparisons between groups were completed using a one-way ANOVA with a Tukey post hoc test for multiple comparisons. ****P < 0.0001.

cGAS∆N mRNA-loaded LNPs showed similar size and mRNA loading profiles to the LNPs loaded with mRNAs encoding the model antigens ovalbumin (OVA) or Green Fluorescent Protein (GFP). LNPs containing mRNAs that code for OVA or GFP are hereafter referred to as antigen-LNPs. All mRNA-loaded LNPs had an average effective diameter of approximately 100 nm (Fig. 1A), with a relatively uniform size profile, exhibited by polydispersity indexes near 0.2 (Fig. 1B). mRNA loading was quantified by measuring the free mRNA in solution and disrupting LNPs to release encapsulated mRNA. Encapsulation efficiency was similar for all mRNAs tested, with approximately 80% of the total mRNA encapsulated in LNPs (Fig. 1C).

### cGAS∆N-LNPs are more immunostimulatory than antigen-LNPs in a human monocytic cell line

To determine the relative immunostimulatory activities of cGAS∆N-LNPs and antigen-LNPs, LNPs were synthesized to contain mRNA encoding OVA or GFP, or mRNA encoding cGAS∆N. These LNPs were tested individually on THP1-Null2 cells, a cell line derived from THP-1 human monocytic cells. This cell line has been used in studies of innate immunity (24), including the cGAS-STING pathway (25). THP1-Null2 cells were treated with LNPs based on the concentration of mRNA content, corresponding to 1 µg/mL GFP, cGAS∆N, or OVA mRNA. After 24 hours of LNP treatment, THP-1 cell supernatants were measured for the presence of the T cell chemokine IP-10, which is commonly associated with cGAS-STING pathway activity (23). cGAS∆N-LNPs induced IP-10 secretion, whereas antigen-LNPs induced minimal IP-10 secretion (Fig. 1D). cGAS∆N-LNPs also induced the upregulation of T cell costimulatory molecule CD40 expression, as compared to GFP or OVA-LNPs (Fig. 1E). These findings suggest that the nature of the protein encoded by the mRNA in an LNP dictates its adjuvanticity.

### cGAS∆N-LNPs activate inflammatory pathways in human moDCs via cGAS-STING

We further explored the immunostimulatory activities of cGAS∆N-LNPs in human monocyte-derived dendritic cells (moDCs), generated from four different leukopak donors. Monocytes were isolated from leukopaks using a CD14^+^ isolation kit. Monocyte purity was assessed after isolation using flow cytometry to determine the frequency of CD14^+^CD16^−^ classical monocytes. Classical monocyte purity post-isolation was greater than 70% for all donors (Fig. S1). Isolated monocytes were differentiated into moDCs in the presence of recombinant granulocyte-macrophage colony-stimulating factor (GM-CSF) and interleukin-4 (IL-4) (26). After 6 days in culture, the efficiency of moDC differentiation was assessed using flow cytometry to quantify CD209^+^CD11c^+^ live cells. For all donors tested, moDC purity was greater than 90% after differentiation (Fig. S2).

**Fig 2:**
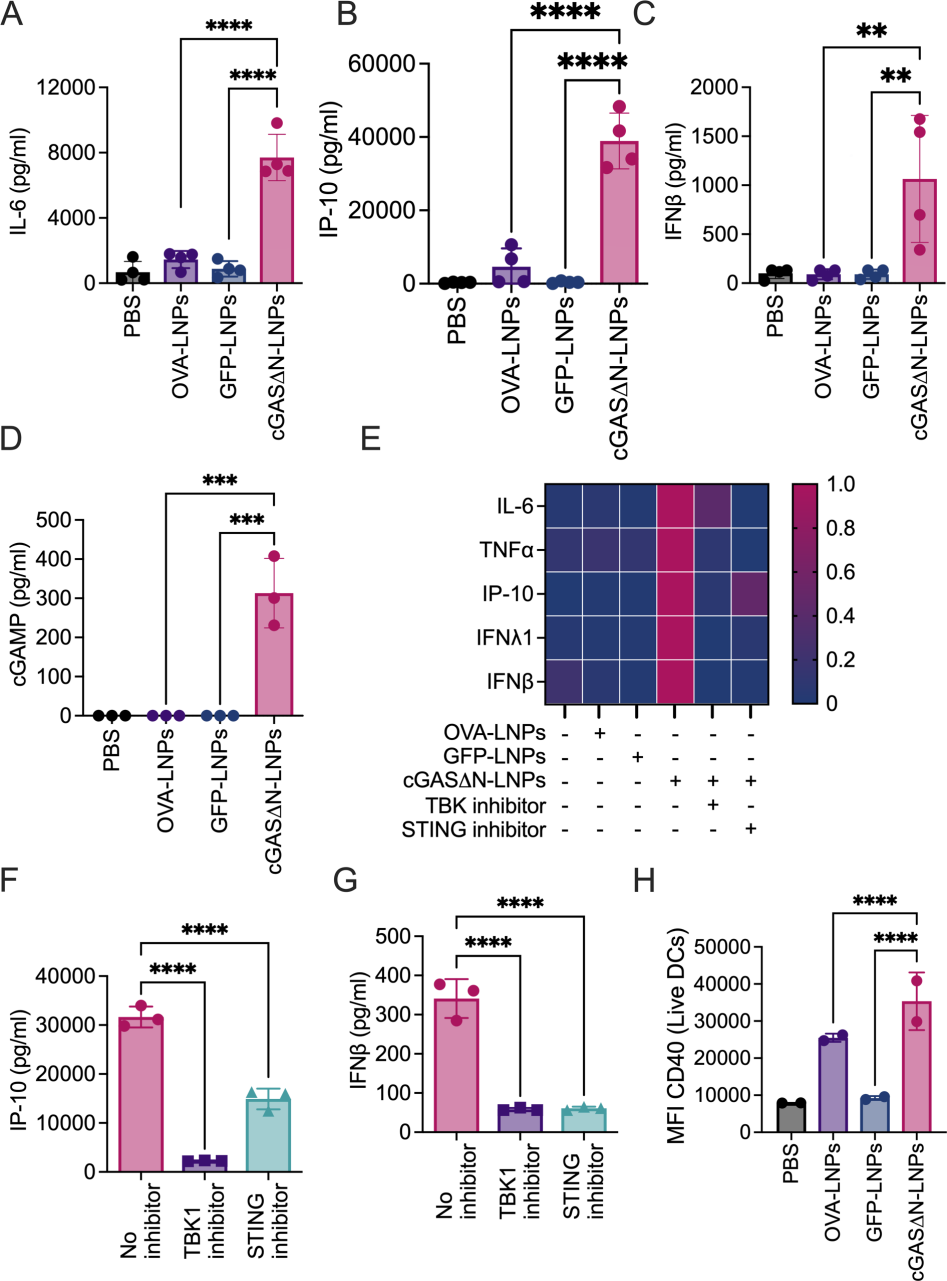
cGAS∆N-LNPs induce human moDC activation. Human moDCs were cultured for 24 hours in the presence of cGAS∆N-LNPs (0.2 µg/mL mRNA in well), GFP-LNPs (0.2 µg/mL mRNA in well), or OVA-LNPs (1.0 µg/mL mRNA in well). (**A**) IL-6, (**B**) IP-10, (**C**) and IFNβ secretion were assessed using a multiplex cytokine bead array. Data on graphs represent the average of a triplicate for each of the four donors. Error bars show the standard deviation between donor cytokine secretion. (**D**) cGAMP production was quantified from cell lysates following LNP treatments. Data represent means and SD of a triplicate from one donor. Data are representative of at least two donors. (**E–H**) MoDCs were cultured in the presence of cGAS∆N-LNPs with or without pre-treatment with TBK1 inhibitor or STING inhibitor for 24 hours. (**E**) Heat map representing normalized cytokine secretion post-treatment of one donor. (**F**) IP-10 and (**G**) IFNβ secretion was measured, and data represent the average of a triplicate from one donor. Data are representative of at least two donors. (**H**) Cells treated for 24 hours in the presence of cGAS∆N-LNPs were stained for activation markers. The MFI of CD40 expression was quantified by flow cytometry. Data represent the MFI for each of the two donors run in triplicate. Error bars show the standard deviation between donor surface marker expressions. Comparisons between groups were completed using a one-way ANOVA with a Tukey post hoc test for multiple comparisons. *P < 0.05, **P < 0.01, ***P < 0.001, and ****P < 0.0001.

MoDCs were treated with LNPs loaded with 0.2 µg/mL GFP or cGAS∆N mRNA, or 1 µg/mL OVA mRNA for 24 hours, then supernatants were collected for cytokine secretion analysis by cytokine bead array assay. cGAS∆N-LNPs induced the production of IL-6, IP-10, and IFNβ (Fig. 2A through C), as well as TNFα and IFNλ1 (Figure S3A and B), whereas these cytokines were poorly expressed by cells treated with LNPs loaded with GFP or OVA mRNA. cGAS∆N immunostimulatory activities result from its enzymatic activities upon cGAS∆N protein production in cells, leading to cGAMP production and downstream STING signaling (17–19). Consistent with this idea, cGAS∆N-LNPs induced cGAMP production from moDCs derived from all healthy donors tested, while LNPs containing OVA or GFP did not (Fig. 2D). To determine whether the activity of cGAS∆N-LNPs was dependent on the STING pathway, moDCs were pre-treated (or not) with two different STING pathway inhibitors: a TBK1 inhibitor (MRT67307) and a STING inhibitor (H-151) 2 hours before LNP treatments. After 24 hours, cytokine secretion was assessed using a multiplexed cytokine bead array assay. TBK1 or STING inhibitor treatments disrupted the ability of cGAS∆N-LNPs to induce the secretion of inflammatory cytokines (distinct human donors assessed in Fig. 2E; Fig. S3C). The reduced cytokine secretion in the presence of the inhibitors was not due to cell death, as the release of lactate dehydrogenase, a common assay for cytolysis, was unaffected by any treatment used in these studies (Fig. S3D). Specifically, we observed that cGAS∆N-induced secretion of IP-10 (Fig. 2F), IFNβ (Fig. 2G), IL-6 (Fig. S3E), TNFα (Fig. S3F), and IFNλ1 (Fig. S3G) was reduced in the presence of TBK1 and STING inhibitors.

The enhanced immunostimulatory activities of cGAS∆N, compared to antigen LNPs, were also evident by flow cytometry for inflammatory surface marker expression on moDCs. Surface marker expression was evaluated on live CD11c^+^CD209^+^ moDCs. CD40, CD80, CD83, and human leukocyte antigen-DR (HLA-DR) expression, quantified by median fluorescence intensity (MFI), was most highly increased when cells were treated with cGAS∆N-LNPs (Fig. 2H and Fig. S4A through C). In the case of MHC class-I molecules, HLA-ABC staining did not reveal cGAS∆N-LNP-dependent changes in surface staining (Fig. S4D). Notably, GFP MFI assessments confirmed that GFP was produced by moDCs treated with GFP-LNPs (Fig. S4E). Coupled with the cytokine and surface marker expression data, these data indicate that antigen expression (e.g., GFP) alone is not sufficient to stimulate inflammatory DC activities. In particular, cGAS∆N-LNPs induced stronger moDC inflammatory responses than antigen-LNPs, even when the antigen-LNPs (OVA) were delivered at a fivefold higher dose (Fig. 2A through C).

### cGAS∆N-LNPs induce STING-dependent type I IFN response in murine DCs

To determine whether the superior immunogenicity of cGAS∆N-LNPs extends to murine cells, DCs were differentiated from the bone marrow of mice using recombinant Fms-like tyrosine kinase 3 ligand (FLT3L). FLT3L generates conventional DCs (cDCs) that are divided into two major subsets: cDC1s and cDC2s (27). Of these subsets, cDC1s are uniquely capable of antigen cross-presentation and can prime naïve CD8^+^ T cells, as well as CD4^+^ T cells (28). Nine days post-differentiation, the efficiency of conventional DC (cDC) differentiation was assessed by flow cytometry as CD11c^+^CD45R^neg^MHC-II^+^live cells, which accounted for at least 78% of the culture (Fig. S5A). Among those cDCs, 54% of cDC1, defined as CD24^+^SIRP1α^neg^, were present in the culture (Fig. S5A).

**Fig 3:**
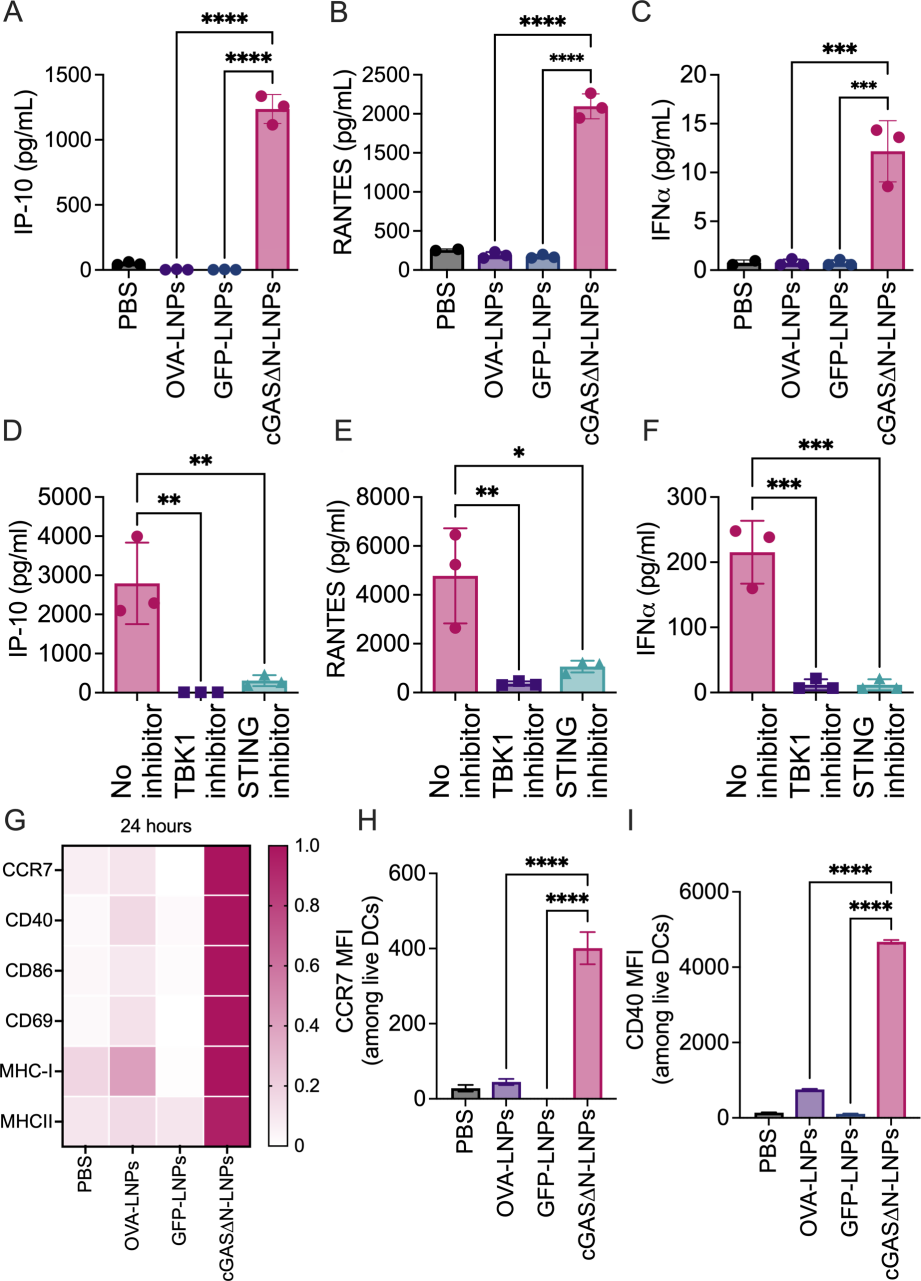
cGAS∆N-LNPs induce type I IFN response and DC activation. (**A–C**) FLT3L-DCs treated with PBS, OVA-LNPs, GFP-LNPs, or cGAS∆N-LNPs. The levels of (**A**) IP-10, (**B**) RANTES, and (**C**) IFNα in the culture supernatant were measured by multiplex bead array. The mean values and SD of three biological triplicates are shown. (**D–F**) FLT3L-DCs were treated with cGAS∆N-LNPs alone or cGAS∆N-LNPs in the presence of a TBK1 inhibitor or STING inhibitor. The levels of (**D**) IP-10, (**E**) RANTES, and (**F**) IFNα in the culture supernatant were measured in triplicates, and the mean values are shown. (**G and H**) FLT3L-DC treated with indicated conditions. The expression of DC activation markers, CCR7, CD40, CD86, CD69, MHC-I, and MHC-II by flow cytometry (**G–I**). The normalized MFI values of each marker are represented as a single color gradient heat map. The MFI and SEM of CCR7 (**H**) and CD40 (**I**) from triplicates are shown. In all the plots, individual data points represent the values of individual treatments of the triplicate. Data are representative of at least two experiments. Comparisons between groups were completed using a one-way ANOVA with a Tukey post hoc test for multiple comparisons. *P < 0.05, **P < 0.01, ***P < 0.005, and ****P < 0.0001.

The bulk population of FLT3L-DCs (containing both cDC1 and cDC2 subsets) was stimulated for 24 hours with phosphate-buffered saline (PBS) or distinct LNPs containing 0.2 µg/mL GFP, cGAS∆N, or OVA mRNA, after which supernatants were collected. Supernatant cytokine abundance was then measured for IP-10, RANTES, and IFNα, all of which are associated with cGAS-STING signaling (23). DCs that were treated with PBS, OVA-LNPs, or GFP-LNPs did not induce IP-10, RANTES, or IFNα secretion, whereas cGAS∆N-LNPs induced the secretion of robust amounts of these cytokines (Fig. 3A through C). All activities induced in DCs by cGAS∆N-LNPs were dependent on the STING pathway, as pre-treatment of cells with TBK1 or STING inhibitors abrogated the secretion of IP-10, RANTES, and IFNα (Fig. 3D through F). These data indicate that, as in human moDCs, cGAS∆N activates STING- and TBK1-dependent IFN activities in FLT3L-DCs.

### cGAS∆N-LNPs induce diverse immunostimulatory activities in murine DCs

To assess whether DC activities other than cytokine production are enhanced by cGAS∆N, FLT3L-DCs were examined for the expression of T cell costimulatory molecules, MHC molecules, and markers of cell migration. 24 hours after LNP treatment, cDCs were stained for CD40, CD86, and CD69 and the mean fluorescence intensity of these costimulatory molecules was measured by flow cytometry (Fig. 3G). The expression of the chemokine receptor CCR7 was upregulated on cDCs when they were treated with cGAS∆N-LNPs, as compared to DCs treated with PBS, OVA-LNPs, or GFP-LNPs (Fig. 3G and H). The expression of T cell costimulatory surface molecules CD40, CD86, and CD69 was also upregulated when cells were treated with cGAS∆N-LNPs, but not with GFP-LNPs or OVA containing LNPs (Fig. 3G and I; Fig. S5B and C). In addition, MHC-I and MHC-II molecules were increased on cDCs treated with cGAS∆N-LNPs, as compared to antigen-LNPs (Fig. 3G; Fig. S5D and E). GFP MFI assessments confirmed that GFP was made by cDCs treated with GFP-LNPs (Fig. S5F), confirming that antigen expression alone is not sufficient to stimulate inflammatory DC activities. Overall, these data indicate that cGAS∆N-LNPs induce several activities necessary for DCs to stimulate T cell responses. These activities include the following: (i) the upregulation of molecules necessary for antigen presentation (and cross-presentation) to T cells; (ii) the upregulation of chemokine receptors and costimulatory molecules necessary for DC migration and interaction with T cells; and (iii) the production of type I IFN-related cytokines that are important to stimulate effector and memory CD8^+^ T cell responses.

### Immunization with antigen-LNPs and cGAS∆N generates durable OVA-specific CD8^+^ T cells

**Fig 4:**
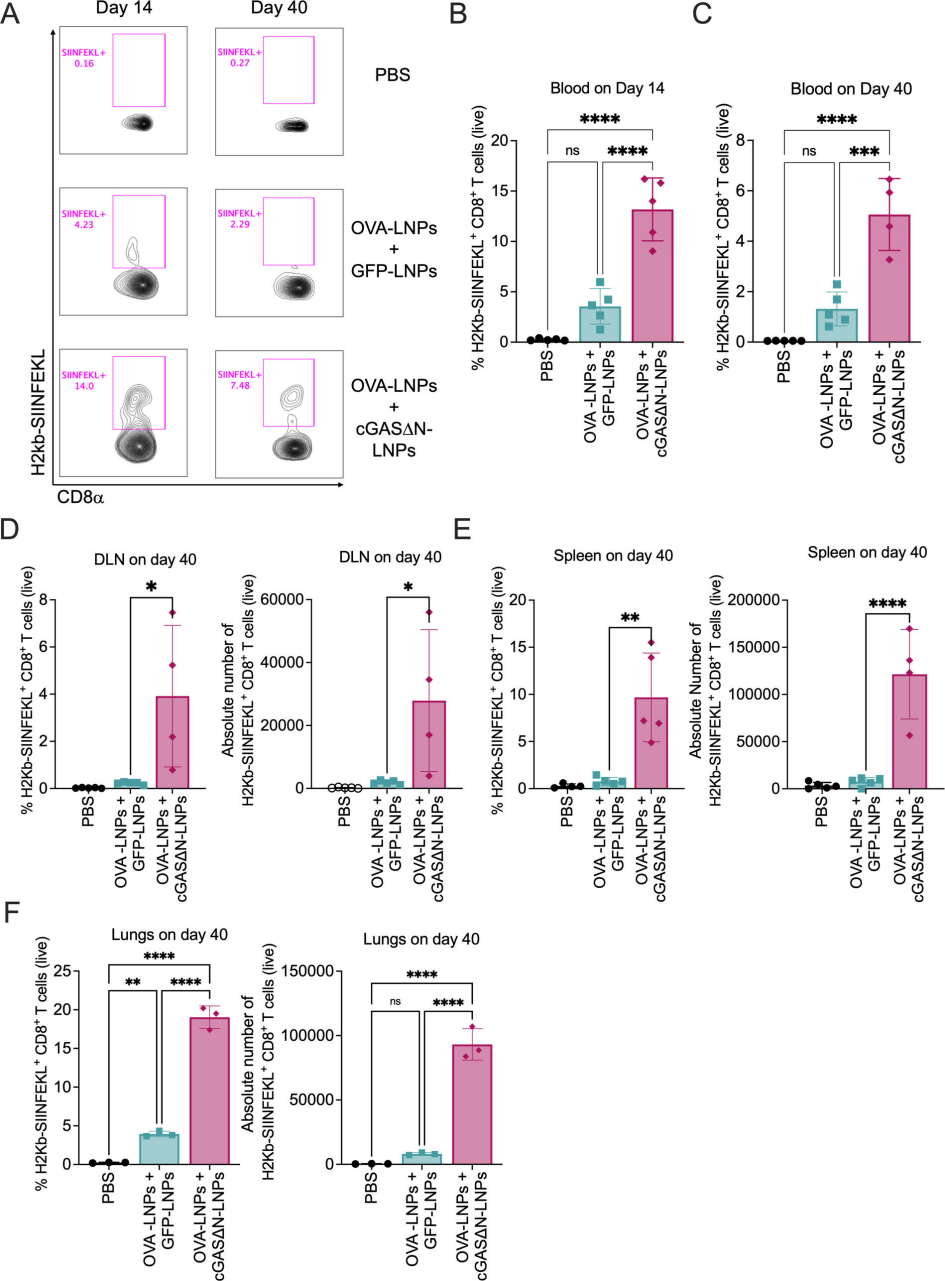
Immunization with antigen-LNPs and cGAS∆N-LNPs induces durable OVA-specific CD8^+^ T cells. (**A–F**) C57BL/6 mice were immunized with PBS or OVA-LNPs in combination with GFP-LNPs or cGAS∆N-LNPs for two injections separated by 7 days. The percentage and absolute number of OVA-specific CD8^+^ T cells were assessed 14 days and 40 days post-immunization by flow cytometry using H2Kb-SIINFEKL tetramer. (**A**) A representative flow cytometry plot of the percentage of H2Kb-SIINFEKL^+^ CD8^+^ T cells is shown. (**B**) The percentage of H2Kb-SIINFEKL^+^ CD8^+^ T cells in the blood was analyzed 14 days (**B**) and 40 days (**C**) after the first immunization. (**D–F**) The percentage (left panel) and absolute number (right panel) of H2Kb-SIINFEKL^+^ CD8^+^ T cells in the (**D**) dLN, (**E**) spleen, (**F**) and lungs were analyzed 40 days after the first immunization *n* = 4–5 mice per group. Samples with low viability were excluded from post-tissue dissociation. The immune cells from the lung tissue were pooled and analyzed in triplicate. Comparisons between groups were completed using a one-way ANOVA with a Tukey post hoc test for multiple comparisons. *P < 0.05, **P < 0.01, ***P < 0.005, and ****P < 0.0001.

To determine whether cGAS∆N-LNPs can modulate antigen-specific T cell responses *in vivo*, OVA was used as a model antigen. The use of OVA allowed tracking of OVA-specific T cell generation using an MHC tetramer that detects CD8^+^ T cells specific to the OVA peptide SIINFEKL (also known as OVA 257–264). As such, mice were injected subcutaneously (s.c.) with PBS or were co-immunized with either OVA-LNPs and cGAS∆N-LNPs, or with OVA-LNPs and GFP-LNPs. Seven days later, mice received a boost injection with the same dose of LNPs. Blood was collected during the effector (14 days post-immunization) and memory (40 days post-immunization) T cell phases. OVA-specific CD8^+^ T cell responses were then assessed by flow cytometry using H2Kb-SIINFEKL tetramers. Fourteen days post-immunization, we found that when mice were co-injected with OVA-LNPs and GFP-LNPs, the frequency of H2Kb-SIINFEKL^+^ CD8^+^ T cells increased, as compared to PBS injection (Fig. 4A and B). However, this frequency was exceeded when mice were co-injected with OVA-LNPs and cGAS∆N-LNPs (Fig. 4A and B). Similar trends were observed 40 days post-immunization (Fig. 4A and C). Mice that were co-injected with OVA-LNPs and GFP-LNPs induced higher amounts of H2Kb-SIINFEKL^+^ CD8^+^ T cells than mice injected with PBS. However, the enhancement of antigen-specific T cell generation at the memory phase was highest in mice that were co-injected with cGAS∆N-LNPs and OVA-LNPs. Similar observations were made when H2Kb-SIINFEKL^+^ CD8^+^ T cells were measured in the lymph nodes that drained injection sites (Fig. 4D), as well as the spleen (Fig. 4E) and lungs (Fig. 4F) 40 days post-immunization. In these tissues, the frequency and absolute number of H2Kb-SIINFEKL^+^ CD8^+^ T cells were highest in mice that were co-injected with OVA-LNPs and cGAS∆N-LNPs (Fig. 4D through F). These data reveal cGAS∆N-LNPs as effective inducers of antigen-specific T cells that are durable and circulating through the lymphatics, blood, and lungs.

### cGAS∆N-LNPs enhance antigen-specific IFNγ production by memory T cells

**Fig 5:**
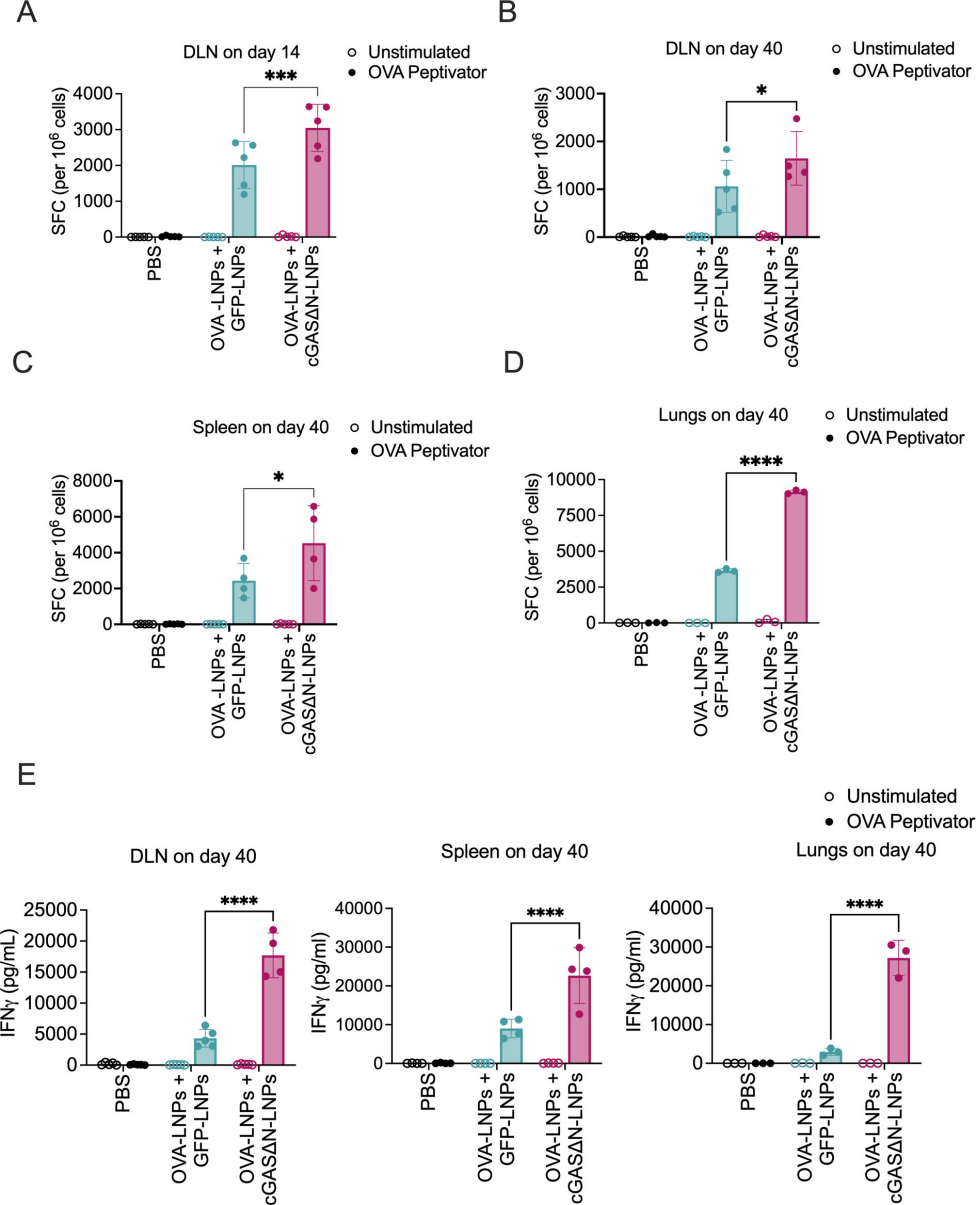
Immunization with antigen-LNPs and cGAS∆N-LNP enhances antigen-specific T cell activity upon antigen re-encounter. (**A–D**) C57BL/6 mice were immunized with PBS or OVA-LNPs in combination with GFP-LNPs or cGAS∆N-LNPs for two injections separated by 7 days. dLN cells from C57BL/6 mice that were immunized with indicated LNPs were harvested on day (**A**) 14 and (**B**) day 40 post-immunization. Single-cell suspensions were re-stimulated with media or OVA peptivator. IFNγ secretion was measured 18 hours post-restimulation using an ELISpot assay. 40 days post-immunization, single-cell suspensions from the (**C**) spleen and (**D**) lungs were re-stimulated for 18 hours with media or OVA peptivator and analyzed for IFNγ secretion by ELISpot. The number of spot-forming cells (SFC) per 10^6^ cells was counted and mean values with SD are shown. (**E**) IFNγ secretion from the dLN (left panel), spleen (middle panel), and lungs (right panel) was measured 96 hours post-restimulation by IFNγ Lumit immunoassay (*n* = 4–5 mice per group). Samples with low viability were excluded from post-tissue dissociation. The cells derived from the lungs were pooled and analyzed in triplicate. Comparisons between groups were completed using a one-way ANOVA with a Tukey post hoc test for multiple comparisons. *P < 0.05, ***P < 0.005, and ****P < 0.0001.

To further assess the specificity and functionality of individual T cells that result from the s.c. immunization with cGAS∆N-LNPs, the skin dLNs from injected mice were harvested 14 and 40 days post-immunization and processed to single-cell suspensions. IFNγ production was then assessed after stimulation (or not) with an OVA peptide (peptivator) library by enzyme-linked immunosorbent spot (ELISpot) assay. cGAS∆N-LNP immunizations enhanced the generation of IFNγ producing T cells in the dLN, as assessed by ELISpot, as compared to mice co-injected with OVA-LNPs and GFP-LNPs (Fig. 5A). Similar results were observed 40 days post-immunization in the dLN, spleen, and lungs of immunized mice (Fig. 5B through D). Furthermore, cGAS∆N-LNPs enhanced IFNγ secretion when single-cell suspension from the dLN, spleen, and lungs of immunized mice were restimulated *ex vivo* with OVA peptivator for 96 hours (Fig. 5E). These data indicate that cGAS∆N-LNPs induce long-lived, antigen-specific, circulating memory T cells that produce high amounts of IFNγ upon antigen re-encounter.

### cGAS∆N-LNPs induce type I biased antigen-specific antibody responses

**Fig 6:**
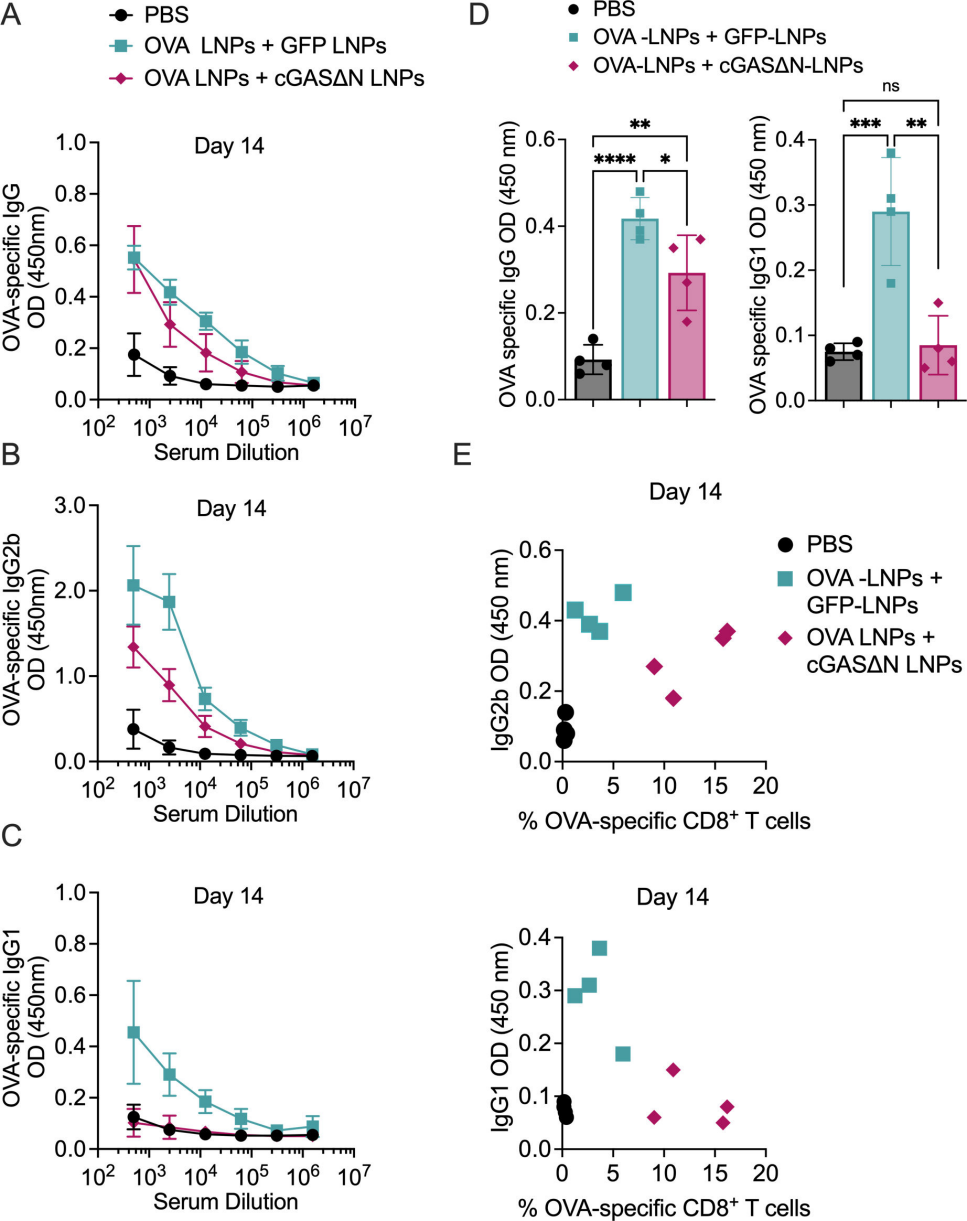
Immunization with antigen-LNPs and cGAS∆N-LNPs elicits an antibody response skewed toward type I immunity 14 days post-immunization. (**A–J**) C57BL/6 mice were immunized with PBS or OVA-LNPs in combination with GFP-LNPs or cGAS∆N-LNPs for two injections separated by 7 days. The serum from immunized mice was analyzed to measure (**A**) OVA-specific IgG, (**B**) IgG2b, and (**C**) IgG1 14 days post-immunization by ELISA. (**D**) The mean OD values for the endpoint titer for OVA-specific IgG as well as OVA-specific IgG1 14 days post-immunization are shown. (**E**) The titer for OVA-specific IgG1 and IgG2b values versus the percent of OVA-specific CD8^+^ T cells in blood 14 days post-immunization are represented as a scatter plot. Each data point represents the value from each mouse. Comparisons between groups were completed using a one-way ANOVA with a Tukey post hoc test for multiple comparisons. *P < 0.05, **P < 0.01, ***P < 0.005, and ****P < 0.0001.

**Fig 7:**
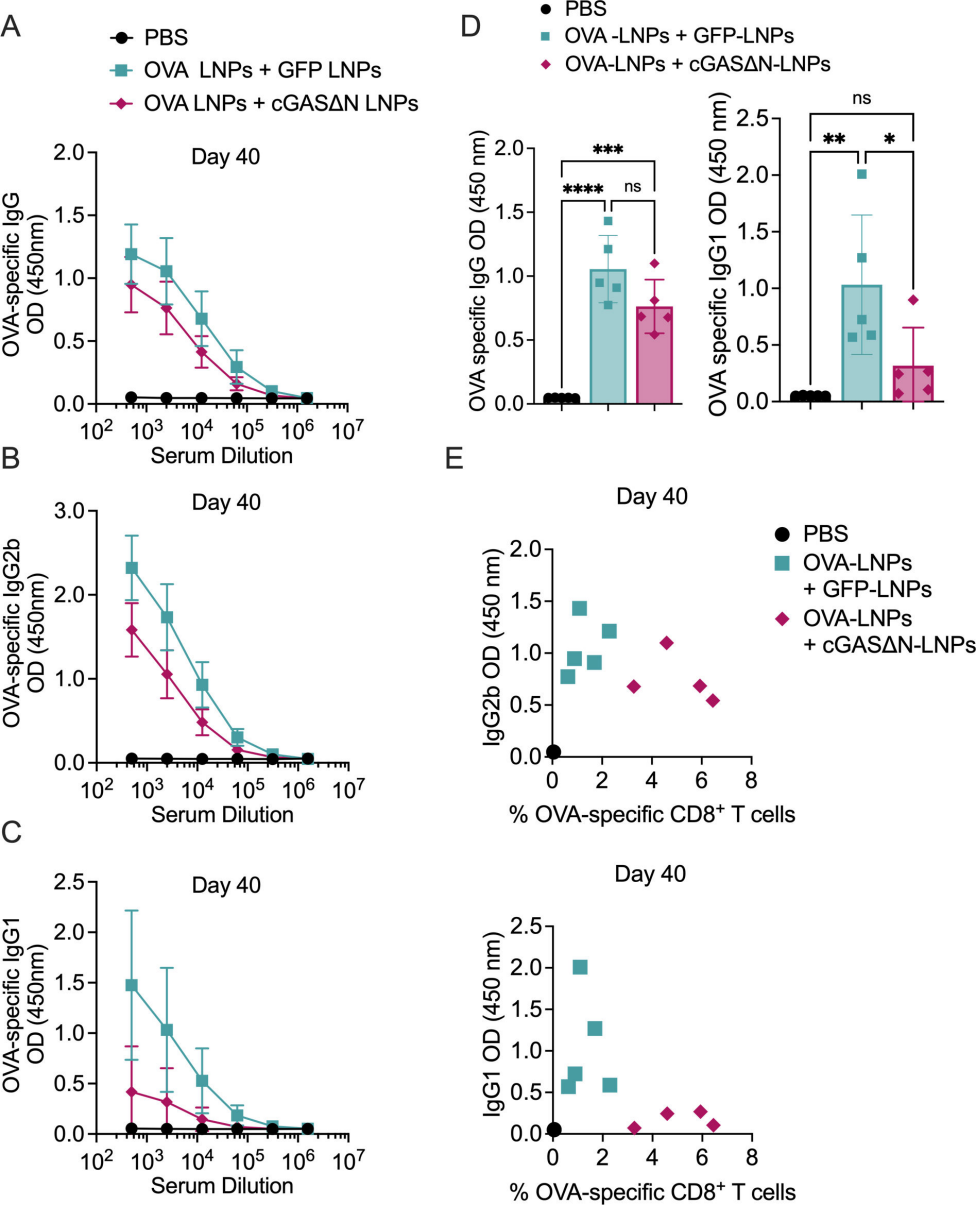
Immunization with antigen-LNPs and cGAS∆N-LNPs elicits an antibody response skewed toward type I immunity 40 days post-immunization. (**A–E**) C57BL/6 mice were immunized with PBS or OVA-LNPs in combination with GFP-LNPs or cGAS∆N-LNPs for two injections separated by 7 days. The serum from immunized mice was analyzed to measure (**A**) OVA-specific IgG, (**B**) IgG2b, and (**C**) IgG1 40 days post-immunization by ELISA. (**D**) The mean OD values for the endpoint titer for OVA-specific IgG as well as OVA-specific IgG1 40 days post-immunization are shown. (**E**) The titer for OVA-specific IgG1 and IgG2b values versus the percent of OVA-specific CD8^+^ T cells in blood 40 days post-immunization are represented as a scatter plot. Each data point represents the value from each mouse. Comparisons between groups were completed using a one-way ANOVA with a Tukey post hoc test for multiple comparisons. *P < 0.05, **P < 0.01, ***P < 0.005, and ****P < 0.0001.

Serum from immunized mice was isolated 14 and 40 days post-immunization to measure OVA-specific IgG, IgG1 (associated with Th2 responses), and IgG2b (associated with Th1 responses) antibody isotypes. Fourteen days post-immunization, mice co-injected with OVA-LNPs and GFP-LNPs induced strong OVA-specific antibody responses, as assessed by the high amounts of OVA-specific IgG, IgG1, and IgG2b detected (Fig. 6A through C), as compared to mice injected with PBS. Mice co-injected with OVA-LNPs and cGAS∆N-LNPs also induced high OVA-specific IgG, as compared to PBS-injected mice (Fig. 6A). cGAS∆N-LNPs induced antibody responses that were biased toward type I isotypes. While the co-injection of OVA-LNPs and cGAS∆N-LNPs slightly reduced IgG2b levels compared to the immunization with OVA-LNPs, OVA-specific IgG1 was completely inhibited by cGAS∆N-LNPs, and were at levels comparable to PBS-injected mice (Fig. 6B through D). The slight reduction of IgG2b levels associated with co-injection of OVA-LNPs and cGAS∆N-LNPs was transient and was not observed 40 days post-immunization, as described below. Assessments of T and B cell responses in the same mice revealed that while co-immunization with OVA-LNPs and GFP-LNPs induced antibody responses that were concomitant with weak T cells, the co-immunization of mice with cGAS∆N-LNPs induced strong antigen-specific CD8^+^ T cell and type I antibody responses (Fig. 6E). These responses were maintained for at least 40 days post-immunization. While mice co-injected with OVA-LNPs and GFP-LNPs induced strong OVA-specific IgG, IgG1, and IgG2b, mice co-injected with OVA-LNPs and cGAS∆N-LNPs induced antibody responses that were biased toward type I isotypes (Fig. 7A through C). The co-injection of OVA-LNPs and cGAS∆N-LNPs induced similar levels of IgG antibodies as OVA-LNPs and GFP-LNPs (Fig. 7D, left panel), whereas cGAS∆N-LNPs prevented OVA-specific IgG1 production (associated with Th2 responses) (Fig. 7D, right panel). Side-by-side comparison of B and T cell responses in mice 40 days post-immunization revealed that cGAS∆N-LNPs strongly induced OVA-specific CD8^+^ T cells and OVA-specific IgG2b antibodies while inhibiting IgG1 responses (Fig. 7E). cGAS∆N-LNPs therefore bolster the antigen-specific T and Th1-biased B cell responses when co-injected with antigen-LNPs.

## DISCUSSION

The cGAS-STING pathway is important for immunity against numerous infections and cancers (29) and has emerged as an attractive target for immunotherapy (30). Efforts are being devoted to develop therapeutic STING modulators including synthetic and natural cyclic dinucleotide (CDN) analogs, as well as non-nucleotidyl small molecule STING agonists that function by mimicking a native ligand of STING, 2′3′ cGAMP (31). However, the therapeutic potential of STING pathway agonists has yet to be reached and limitations associated with current technologies have narrowed their therapeutic potential. For example, exogenous delivery to cells or animals of STING agonists suffers from poor pharmacokinetic and physiochemical properties of these modulators, as STING agonists must penetrate the cell membrane to exert their effects. In addition, natural CDNs are large in size, electronegative, hydrophilic, and susceptible to enzymatic degradation by phosphodiesterases, primarily ENPP1 (32), leading to low drug bioavailability in tissues (30). To overcome these limitations, we explored in this study an alternative approach to STING agonism. Rather than delivering STING ligands through exogenous routes, we took advantage of the enzymatic nature of cGAS to produce STING ligands within cells. This idea was tested through the use of cGASΔN-mRNAs encapsulated in LNPs, which act as a catalytic adjuvant. cGASΔN-LNPs produced 2′3′ cGAMP that potently activates the STING pathway in DCs. In addition, when combined with antigen-LNPs, cGASΔN-LNPs induced durable systemic antigen-specific responses.

By extending the concept of using mRNAs that encode antigens in LNPs to mRNAs that encode protein adjuvants in LNPs, several opportunities arise to enhance the immunogenicity of current vaccines. It remains challenging to develop protective vaccines that improve cellular immunity mediated by T cells while inducing a strong humoral response (33). Indeed, while current mRNA-LNP COVID-19 vaccines are effective at inducing antigen production, these LNPs display weak adjuvant activity (15, 16). Our finding that mRNA encoding the immunostimulatory protein human cGASΔN encapsulated in LNPs preserves antigen production while acting as a catalytic adjuvant to enhance the immunogenicity of antigens may prove useful in the aforementioned contexts. These data provide the mandate to further explore the efficacy of cGASΔN-LNPs combined with antigen-LNPs in protection against pathogens or cancer. Furthermore, future attempts to incorporate both cGASΔN mRNA and antigen mRNA in the same LNP to enhance the delivery of both adjuvant and antigen to the same DC may prove effective at providing protection in infectious and non-infectious challenge models. Our study therefore opens new avenues to explore the use of mRNA encoding immune signaling proteins in LNPs, rather than antigens alone, which when combined can induce cell-mediated immunity that ensures durable host defense.

## MATERIALS AND METHODS

### LNP synthesis

Lipids for LNP synthesis were acquired from Caymen Chemicals {8-[(2-hydroxyethyl)[6-oxo-6-(undecyloxy)hexyl]amino]-octanoic acid, 1-octylnonyl ester(SM-102)} and Avanti (distearoyl-phosphatidylcholine[DSPC]; 1,2-dimyristoyl-rac-glycero-3-methoxypolyethylene glycol-2000 [DMG-PEG2000]). Cholesterol was purchased from Sigma. mRNAs for LNP synthesis were purchased from TriLink BioTechnologies. CleanCap mRNAs encoding ovalbumin (OVA) and enhanced green fluorescent protein (GFP) modified with 5-methoxyuridine (5moU) were purchased off-the-shelf (TriLink) (Table 1). cGAS∆N mRNA was custom-ordered and synthesized via *in vitro* transcription from linearized template DNA (Table 1). The nucleotide sequence of the human cGAS∆N was Condon optimized for expression in murine cells. The mRNA sequence was capped using Trilink’s CleanCap mRNA technology, with an N1-methylpseudouridine base modification, and a 120 residue polyA tail. The sequence contains a Bbsl restriction enzyme site. The mRNA was phosphatase treated after synthesis.

**TABLE 1:** mRNA sequence used

mRNA sequence for:	mRNA sequence	Vendor	Catalog number
CleanCap Ovalbumin mRNA (5-methoxyuridine)	AUGGGCAGCAUCGGCGCCGCCAGCAUGGAGUUCUGCUUCGACGUGUUCAAGGAGCUGAAGGUGCACCACGCCAACGAGAACAUCUUCUACUGCCCCAUCGCCAUCAUGAGCGCCCUGGCCAUGGUGUACCUGGGCGCCAAGGACAGCACCCGGACCCAGAUCAACAAGGUGGUGCGGUUCGACAAGCUGCCCGGCUUCGGCGACAGCAUCGAGGCCCAGUGCGGCACCAGCGUGAACGUGCACAGCAGCCUGCGGGACAUCCUGAACCAGAUCACCAAGCCCAACGACGUGUACAGCUUCAGCCUGGCCAGCCGGCUGUACGCCGAGGAGCGGUACCCCAUCCUGCCCGAGUACCUGCAGUGCGUGAAGGAGCUGUACCGGGGCGGCCUGGAGCCCAUCAACUUCCAGACCGCCGCCGACCAGGCCCGGGAGCUGAUCAACAGCUGGGUGGAGAGCCAGACCAACGGCAUCAUCCGGAACGUGCUGCAGCCCAGCAGCGUGGACAGCCAGACCGCCAUGGUGCUGGUGAACGCCAUCGUGUUCAAGGGCCUGUGGGAGAAGACCUUCAAGGACGAGGACACCCAGGCCAUGCCCUUCCGGGUGACCGAGCAGGAGAGCAAGCCCGUGCAGAUGAUGUACCAGAUCGGCCUGUUCCGGGUGGCCAGCAUGGCCAGCGAGAAGAUGAAGAUCCUGGAGCUGCCCUUCGCCAGCGGCACCAUGAGCAUGCUGGUGCUGCUGCCCGACGAGGUGAGCGGCCUGGAGCAGCUGGAGAGCAUCAUCAACUUCGAGAAGCUGACCGAGUGGACCAGCAGCAACGUGAUGGAGGAGCGGAAGAUCAAGGUGUACCUGCCCCGGAUGAAGAUGGAGGAGAAGUACAACCUGACCAGCGUGCUGAUGGCCAUGGGCAUCACCGACGUGUUCAGCAGCAGCGCCAACCUGAGCGGCAUCAGCAGCGCCGAGAGCCUGAAGAUCAGCCAGGCCGUGCACGCCGCCCACGCCGAGAUCAACGAGGCCGGCCGGGAGGUGGUGGGCAGCGCCGAGGCCGGCGUGGACGCCGCCAGCGUGAGCGAGGAGUUCCGGGCCGACCACCCCUUCCUGUUCUGCAUCAAGCACAUCGCCACCAACGCCGUGCUGUUCUUCGGCCGGUGCGUGAGCCCCUGA	TriLink	L-7210
CleanCap EGFP mRNA (5moU)	AUGGUGAGCAAGGGCGAGGAGCUGUUCACCGGGGUGGUGCCCAUCCUGGUCGAGCUGGACGGCGACGUAAACGGCCACAAGUUCAGCGUGUCCGGCGAGGGCGAGGGCGAUGCCACCUACGGCAAGCUGACCCUGAAGUUCAUCUGCACCACCGGCAAGCUGCCCGUGCCCUGGCCCACCCUCGUGACCACCCUGACCUACGGCGUGCAGUGCUUCAGCCGCUACCCCGACCACAUGAAGCAGCACGACUUCUUCAAGUCCGCCAUGCCCGAAGGCUACGUCCAGGAGCGCACCAUCUUCUUCAAGGACGACGGCAACUACAAGACCCGCGCCGAGGUGAAGUUCGAGGGCGACACCCUGGUGAACCGCAUCGAGCUGAAGGGCAUCGACUUCAAGGAGGACGGCAACAUCCUGGGGCACAAGCUGGAGUACAACUACAACAGCCACAACGUCUAUAUCAUGGCCGACAAGCAGAAGAACGGCAUCAAGGUGAACUUCAAGAUCCGCCACAACAUCGAGGACGGCAGCGUGCAGCUCGCCGACCACUACCAGCAGAACACCCCCAUCGGCGACGGCCCCGUGCUGCUGCCCGACAACCACUACCUGAGCACCCAGUCCGCCCUGAGCAAAGACCCCAACGAGAAGCGCGAUCACAUGGUCCUGCUGGAGUUCGUGACCGCCGCCGGGAUCACUCUCGGCAUGGACGAGCUGUACAAGUAA	TriLink	L-7201
Human cGASDN mRNA sequence with codon optimization for mouse	AUGCCCGGCGCCAGCAAGCUGAGGGCCGUGCUGGAGAAGCUGAAGCUGAGCAGGGACGACAUCAGCACCGCCGCCGGCAUGGUGAAGGGCGUGGUGGACCACCUGCUGCUGAGGCUGAAGUGCGACAGCGCCUUCAGGGGCGUGGGCCUGCUGAACACCGGCAGCUACUACGAGCACGUGAAGAUCAGCGCCCCCAACGAGUUCGACGUGAUGUUCAAGCUGGAGGUGCCCAGGAUCCAGCUGGAGGAGUACAGCAACACCAGGGCCUACUACUUCGUGAAGUUCAAGAGGAACCCCAAGGAGAACCCCCUGAGCCAGUUCCUGGAGGGCGAGAUCCUGAGCGCCAGCAAGAUGCUGAGCAAGUUCAGGAAGAUCAUCAAGGAGGAGAUCAACGACAUCAAGGACACCGACGUGAUCAUGAAGAGGAAGAGGGGCGGCAGCCCCGCCGUGACCCUGCUGAUCAGCGAGAAGAUCAGCGUGGACAUCACCCUGGCCCUGGAGAGCAAGAGCAGCUGGCCCGCCAGCACCCAGGAGGGCCUGAGGAUCCAGAACUGGCUGAGCGCCAAGGUGAGGAAGCAGCUGAGGCUGAAGCCCUUCUACCUGGUGCCCAAGCACGCCAAGGAGGGCAACGGCUUCCAGGAGGAGACCUGGAGGCUGAGCUUCAGCCACAUCGAGAAGGAGAUCCUGAACAACCACGGCAAGAGCAAGACCUGCUGCGAGAACAAGGAGGAGAAGUGCUGCAGGAAGGACUGCCUGAAGCUGAUGAAGUACCUGCUGGAGCAGCUGAAGGAGAGGUUCAAGGACAAGAAGCACCUGGACAAGUUCAGCAGCUACCACGUGAAGACCGCCUUCUUCCACGUGUGCACCCAGAACCCCCAGGACAGCCAGUGGGACAGGAAGGACCUGGGCCUGUGCUUCGACAACUGCGUGACCUACUUCCUGCAGUGCCUGAGGACCGAGAAGCUGGAGAACUACUUCAUCCCCGAGUUCAACCUGUUCAGCAGCAACCUGAUCGACAAGAGGAGCAAGGAGUUCCUGACCAAGCAGAUCGAGUACGAGAGGAACAACGAGUUCCCCGUGUUCGACGAGUUCUAAUGA	TriLink	N/A

The lipid mix was prepared with 40% SM-102 as ionizable lipid, 30% DSPC, 28.5% cholesterol, and 1.5% DMG-PEG2000. The total lipid concentration used for synthesis was 12.5 mM in ethanol. OVA mRNA, GFP mRNA, and cGAS∆N mRNA were each prepared at 0.17 mg/mL in sodium citrate buffer, pH 4. LNPs were synthesized using the NanoAssemblr Ignite instrument (Precision Nanosystems). Lipids in ethanol were combined with the mRNA solutions individually at a 1:3 volumetric ratio, using a flow rate of 12 mL/min. LNPs were washed in 10 volumes of PBS, pH 7.4 to remove residual ethanol, and then concentrated using Amicon 10K MWCO centrifugal filters. LNPs were filtered through a 0.2-µM filter before use.

### LNP characterization

Loading of mRNA into LNPs was quantified using a RiboGreen assay (ThermoFisher) following the manufacturer’s protocol. Samples were diluted to fall within the range of the standard curve. LNPs were disrupted using Triton X-100 to assess the encapsulation of mRNA into LNPs. Both total mRNA and encapsulated mRNA were quantified. The size of the LNPs was assessed using dynamic light scattering (DLS) on the NanoBrook Omni (Brookhaven). LNPs were diluted 1:10 in PBS before running on the DLS. Four 180-second measurements were recorded for each sample, with the first measurement discarded and the following three measurements used to calculate the size of the LNPs.

### THP-1 cell culture and activation

THP1-Null2 cells were purchased from Invivogen and thawed into and maintained in RPMI 1640 medium containing 10% heat-inactivated fetal bovine serum (FBS), 25 mM HEPES, 100 U/mL penicillin-streptomycin, and 100 µg/mL normocin. Cells were passaged twice a week and maintained between 4E5-6E5 cells/mL in T75 culture flasks. Cell culture media was supplemented with 100 µg/mL zeocin every other passage. Cells were maintained until passage 20, after which a new vial was thawed. Before treating with LNPs, THP1-Null2 cells were collected from flasks and plated in RPMI medium containing 10% heat-inactivated FBS, 25 mM HEPES, and 100 U/mL penicillin-streptomycin at 1E5 cells/well in 96-well flat-bottom tissue culture plates.

THP1-Null2 cells were treated with LNPs based on the concentration of mRNA content. Cells were treated with 1 µg/mL GFP, cGAS∆N, or OVA mRNA delivered in LNPs in a total stimuli volume of 200 µL/well. After an overnight incubation, cells and culture supernatant were used for downstream readouts. An amount of 150 µL of culture supernatant was collected for cytokine secretion analysis, and cells were collected to stain for cell surface activation markers. IP-10 secretion was measured by ELISA (Biolegend). THP1-Null2 cells were stained to measure the expression of CD40. To complete the staining, cells were first washed in PBS to remove the media. Cells were then resuspended in 100 µL PBS containing Live/Dead Near-IR (1:1,000) and incubated for 15 minutes at 4°C. Cells were then washed and resuspended in 100 µL FACS buffer containing Fc block (1:100) for 10 minutes. THP1-Null2 cells were then resuspended in 100 µL of a 1:1 FACS buffer: Brilliant Stain Buffer (BD Biosciences) mixture containing anti-human CD40 (1:200) (Table 2), then incubated for 20 minutes at 4°C. Cells were then washed twice and resuspended in 150 µL FACS buffer, then analyzed on a BD FACS Symphony A3. Each biological condition was tested in triplicate. Data are representative of two experiments.

**TABLE 2:** List of antibodies used^*a*^

Antibody used for:	mAb	Clone	Fluorophore	Vendor	Catalog number
Human monocyte isolation purity	Live/Dead Near-IR	N/A	APC-Cy7 N/A	ThermoFisher	L10119
	Fc block	Fc1	N/A	BD Biosciences	564220
	Anti-human CD14	63D3	APC	Biolegend	367118
	Anti-human CD16	3G8	BUV395	BD Biosciences	563785
Human moDC QC staining	Live/Dead Near-IR	N/A	APC-Cy7	ThermoFisher	L10119
	Fc Block	Fc1	N/A	BD Biosciences	564220
	Anti-human CD11c	B-ly6	FITC	BD Biosciences	561355
	Anti-human CD209 (DC-SIGN)	9E9A8	PE	Biolegend	330106
Human moDC and THP1 activation staining	Live/Dead Near-IR	N/A	APC-Cy7	ThermoFisher	L10119
	Fc Block	Fc1	N/A	BD Biosciences	564220
	Anti-human CD11c	B-ly6	PE-Cy7	BD Biosciences	561356
	Anti-human CD209 (DC-SIGN)	9E9A8	PE	Biolegend	330106
	Anti-human HLA-DR	G46-6	BUV395	BD Biosciences	564040
	Anti-human HLA-ABC	G46-2.6	BV605	BD Biosciences	740407
	Anti-human CD40	5C3	BUV563	BD Biosciences	741381
	Anti-human CD80	2D10.4	BB700	BD Biosciences	751734
	Anti-human CD83	HB15e	BUV737	BD Biosciences	612823
Tetramer staining	Anti-mouse CD3	17A2	BUV395	BD Biosciences	740268
	Anti-mouse CD4	GK1.5	PerCP/ Cyanine5.5	Biolegend	100434
	Anti-mouse CD8a alpha monoclonal antibody	KT15	FITC	Invitrogen	MA5-16759
H2Kb-OVA-specific tetramer	H2Kb-restricted SIINFEKL (OVA 257–264) tetramer	N/A	PE	MBL	TB-5001–1
Murine DC purity	Live/Dead Violet	N/A	V421	ThermoFisher	L34955
	Fc block	2.4G2	N/A	BD Biosciences	553142
	Anti-CD11c	Clone HL3	PE-Cy7	BD Biosciences	558079
	Anti-SIRP1α	P84	FITC	BD Biosciences	560316
	Anti-CD24	M1/69	BV711	BD Biosciences	563450
	Anti-CD45R	RA3-6B2	AF700	BD Biosciences	557957
	Anti-mouse I-A/I-E	2G9	BUV395	BD Biosciences	743876
Murine DC activation analysis	Anti-CD86	PO3	BB700	BD Biosciences	742145
	Anti-CD40	3/23	APC	BioLegend	124612
	Anti-H2-Kb	AF6-88.5	PB	BioLegend	116513
	Anti-CCR7	4B12	PE	BioLegend	120105
	Anti-CD69	H1.2F3	BV605	BD Biosciences	563290

^*a*^ APC = allophycocyanin, AF = Alexa Fluor, Cy = cyanine, BUV = BD Horizon Brilliant Ultraviolet, BV = Brilliant Violet, BB = BD Horizon Brilliant Blue, FITC = fluorescein isothiocyanate, PB = Pacific Blue, PE = phycoerythrin, PerCP = peridinin-chlorophyll-protein, PE-Cy = phycoerythrin-cyanine, N/A = not applicable.

### Human moDC generation

Human monocytes were isolated from Leukopaks purchased from Miltenyi using the StraightFrom Leukopak CD14^+^ microbead kit (Miltenyi). Isolations were completed following the manufacturer’s instructions. Monocytes were then aliquoted and frozen in fetal bovine serum (Gibco) containing 10% dimethyl sulfoxide (ThermoScientific). Monocyte purity was assessed by flow cytometry. Monocytes (<100,000/well) were spun at 400 × *g* for 4 minutes and then washed with PBS once before cell staining. Cells were then resuspended in 100 µL PBS containing Live/Dead Near-IR (1:1,000) and incubated for 15 minutes at 4°C. Cells were then washed and resuspended in 100 µL FACS buffer containing Fc block (1:200) for 10 minutes. Monocytes were then resuspended in 100 µL of a 1:1 FACS buffer: Brilliant Stain Buffer (BD Biosciences) mixture containing anti-human CD14 (1:200) and anti-human CD16 (1:200) (Table 2) and incubated for 20 minutes at 4°C. Cells were then washed twice and resuspended in 150 µL FACS buffer, then analyzed on a BD FACS Symphony A3.

For studies with moDC cultures, monocytes were thawed and cultured in R10 media: RPMI medium (Gibco) supplemented with 10% FBS, 100 U/mL penicillin-streptomycin (Gibco), 2 mM L-glutamine (Gibco), 1 mM sodium pyruvate (Gibco), 10 mM HEPES (Gibco), 55 µM beta-mercaptoethanol, and 1× MEM non-essential amino acids (Gibco). To differentiate monocytes into moDCs, 50 ng/mL recombinant human GM-CSF (Miltenyi) and 25 ng/mL recombinant human IL-4 (Miltenyi) were added to R10 media. Cells were cultured for 6 days with GM-CSF and IL-4, with an additional cell feeding with R10 media containing GM-CSF and IL-4 on day 3. Six days after differentiation, moDCs were collected and counted. Cells were plated into 96-well flat-bottom tissue culture plates at 1E5 cells/well in R10 media.

MoDC purity was assessed by flow cytometry. MoDCs (1E5 cells/well) were spun at 400 × *g* for 4 minutes and then washed with PBS once before cell staining. Cells were then resuspended in 100 µL PBS containing Live/Dead Near-IR (1:1,000) and incubated for 15 minutes at 4°C. Cells were then washed and resuspended in 100 µL FACS buffer containing Fc block (1:100) for 10 minutes. MoDCs were then resuspended in 100 µL of a 1:1 FACS buffer: Brilliant Stain Buffer (BD Biosciences) mixture containing anti-human CD11c (1:200), and anti-human CD209 (1:200) (Table 2) and then incubated for 20 minutes at 4°C. Cells were then washed twice and resuspended in 150 µL FACS buffer, then analyzed on a BD FACS Symphony A3.

### Human moDC activations

Human moDCs were treated with LNPs based on the concentration of mRNA content. Cells were treated with 0.2 µg/mL GFP or cGAS∆N mRNA, or 1 µg/mL OVA mRNA delivered in LNPs in a total stimuli volume of 200 µL/well. Four human donors were used for experiments assessing total cytokine secretion in response to cGAS∆N-LNP stimulation. For the two donors that also received STING pathway inhibition, moDCs treated with cGAS∆N-LNPs were also pre-treated with MRT67307 (TBK1 inhibitor, Selleck Chemicals) at 2.5 µM or H-151 (STING inhibitor, Invivogen) at 2 µM for 2 hours before LNP addition.

After an overnight incubation, cells and culture supernatant were used for downstream readouts. For all four donors, 150 µL of culture supernatant was collected for cytokine secretion analysis. For two donors, cells were collected to stain for cell surface activation markers. For the remaining two donors, cells were lysed to detect the production of cGAMP. Viability assessments for lactate dehydrogenase secretion were completed using the CyQuant LDH Cytotoxicity Assay (Invitrogen) according to the manufacturer’s protocol. The percentage of viability was calculated relative to PBS-treated moDCs.

For two donors, cells were collected for surface marker staining for CD11c, CD209, CD40, CD80, CD83, HLA-ABC, and HLA-DR. GFP expression was also assessed. Cells were washed to remove PBS, then resuspended in 100 µL PBS containing Live/Dead Near-IR (1:1,000) and incubated for 20 minutes at 4°C. Cells were then washed and resuspended in 100 µL FACS buffer containing Fc block (1:100) for 10 minutes. Cells were washed and then resuspended in 100 µL of a 1:1 FACS buffer:Brilliant Stain Buffer (BD Biosciences) mixture containing anti-human CD11c (1:200), anti-human CD209 (1:200), anti-human HLA-DR (1:400), anti-human HLA-ABC (1:200), anti-human CD80 (1:200), anti-human CD83 (1:200), anti-human CD40 (1:200)(Table 2) and then incubated for 20 minutes at 4°C. Cells were then washed and resuspended in 100 µL of 4% PFA to fix the cells for 15 minutes at room temperature. After fixation, cells are washed twice with FACS buffer and kept at 4°C overnight in 150 µL FACS buffer. MoDCs were then analyzed using a BD FACS Symphony A3. Graphs show the median and SD of a triplicate for two donors. Data are representative of two experiments.

### Murine bone marrow-derived FLT3L-DCs generation

Leg femur and tibia were removed from mice, cut with scissors, and flushed into sterile tubes. Bone marrow suspension was treated with ACK lysis buffer for 1 minute, then passed through a 40 µM cell strainer. Cells were counted and resuspended in media consisting of complete IMDM containing 10% FBS, 100 U/mL penicillin-streptomycin, 2 mM L-glutamine, and 1 mM sodium pyruvate (I10). Cells were then plated at 8E6 bone marrow cells per well in a 12-well plate. Recombinant mouse FLT3L (Miltenyi) was added to cultures at 200 ng/mL. Differentiated cells were used for subsequent assays on day 8. The efficiency of differentiation was monitored by flow cytometry using a BD Symphony A3, and CD11c^+^MHC-II^+^ cells were routinely above 80% of living cells. For each experiment, 5 to 15 mice were used to generate DCs from bone marrow.

FLT3L-DC purity was assessed by flow cytometry. FLT3L-DCs (1E5 cells/well) were spun at 400 × *g* for 4 minutes and then washed with PBS once before cell staining. Cells were then resuspended in 100 µL PBS containing Live/Dead Violet (1:1,000) and incubated for 20 minutes at 4°C. Cells were then washed and resuspended in 100 µL FACS buffer containing Fc block (1:100) for 10 minutes. FLT3L-DCs were then resuspended in 100 µL of a 1:1 FACS buffer: Brilliant Stain Buffer (BD Biosciences) mixture containing anti-mouse CD11c (1:200), anti-mouse SIRP1α (1:200), anti-mouse CD24 (1:200), anti-mouse CD45R (1:200), and anti-mouse IA/IE (1:200) (Table 2), then incubated for 20 minutes at 4°C. Cells were then washed and resuspended in 100 µL of 4% PFA to fix the cells for 20 minutes at 4°C. After fixation, cells are washed twice with FACS buffer and kept at 4°C overnight in 150 µL FACS buffer. FLT3L-DCs were then analyzed using a BD FACS Symphony A3.

### Murine FLT3L-DCs stimulation

FLT3L-DCs were harvested 9 days post-differentiation, counted, and then plated at 2E5 cells/well in a 96-well plate. DCs were treated with LNPs based on the concentration of mRNA content. Cells were treated with 0.2 µg/mL OVA, GFP, or cGAS∆N mRNA delivered in LNPs in a total stimuli volume of 200 µL/well. For the DCs that also received STING pathway inhibition, FLT3L-DCs treated with cGAS∆N-LNPs were also pre-treated with MRT67307 (TBK1 inhibitor) at 2.5 µM or H-151 (STING inhibitor) at 2 µM for 2 hours before LNP addition.

To assess DC activation post-LNP exposure, FLT3L-DCs were collected 24 hours after LNP addition and stained to measure the expression of the following cell surface activation markers: CD11c, MHC-II, CD24, SIRP1α, CD40, CD86, CD69, and H2Kb. GFP expression was also assessed. FLT3L DC activation was assessed by flow cytometry. FLT3L-DCs (1E5 cells/well) were spun at 400 × *g* for 4 minutes and then washed with PBS once before cell staining. Cells were then resuspended in 100 µL PBS containing Live/Dead Violet (1:1,000) and incubated for 20 minutes at 4°C. Cells were then washed and resuspend in 100 µL FACS buffer containing Fc block (1:100) for 10 minutes. FLT3L-DCs were then resuspended in 100 µL of a 1:1 FACS buffer: Brilliant Stain Buffer (BD Biosciences) mixture containing anti-mouse CD11c (1:200), anti-mouse SIRP1α (1:200), anti-mouse CD24 (1:200), anti-mouse CD45R (1:200), anti-mouse IA/IE (1:200), anti-mouse CD86 (1:200), anti-mouse CD40 (1:200), anti-mouse H2Kb (1:200), anti-mouse CD69 (1:200), and anti-mouse CCR7 (1:200) (Table 2), then incubated for 20 minutes at 4°C. Cells were then washed and resuspended in 100 µL of 4% PFA to fix the cells for 20 minutes at 4°C. After fixation, cells are washed twice with FACS buffer and kept at 4°C overnight in 150 µL FACS buffer. FLT3L-DCs were then analyzed using a BD FACS Symphony A3. Graphs show the median and SD of a triplicate. Data are representative of two experiments.

### cGAMP ELISA

Cells were lysed to collect cGAMP after 24 hours in culture, using a Triton X-100-based extraction buffer for 10 minutes at room temperature. Cell debris was pelleted by spinning at 400 × *g* for 4 minutes. Supernatants were then collected to perform a competitive ELISA for cGAMP (Cayman Chemicals) according to the manufacturer’s instructions. cGAMP was quantified for two human donors.

### Multiplex measurement of cytokines

Secreted cytokines were measured in the supernatant by cytokine bead array using the LEGENDplex mouse and Human Anti-Virus Response Panel (Biolegend) according to the manufacturer’s protocol. Data were collected using a Quanteon Novocyte flow cytometer and analyzed using the cloud-based software provided by Biolegend.

### Mouse strains and experimental procedures

Eight- to twelve-week-old C57BL/6J mice were purchased from Jackson Labs. Mice were allowed to acclimate to the Explora BioLabs housing facility for at least 1 week. In all experiments, mice were randomly assigned to experimental groups. All experimental procedures were approved by the institutional animal care and use committee at Explora BioLabs (Protocol ID: EB17-010-300).

### *In vivo* immunization with LNPs containing cGAS∆N mRNA and OVA antigen mRNA

C57BL/6J mice were immunized subcutaneously with LNPs. Antigen was delivered using OVA mRNA-loaded LNPs, as well as LNPs containing either the catalytic adjuvant cGAS∆N mRNA or a control GFP mRNA-loaded LNPs. Mice were injected with 5 µg mRNA/mouse. Mice were given a primary immunization on day 0, followed by a boost immunization of the same mRNA-LNPs dose on day 7.

### Blood collection and processing

Mice were placed under isoflurane for approximately 10 minutes for anesthesia. Using a 21G needle, mice were gently poked through the skin to the submandibular space to induce bleeding. Five to six drops were collected in a mini-collect K2EDTA blood collection tube for tetramer staining, or two to three drops were collected in a serum separation tube for antibody assessments. Blood samples were transported back to Corner Therapeutics lab at room temperature (RT) and were allowed to warm up to RT for at least 15 minutes.

To process the blood for tetramer staining, 1 mL of RBC lysis buffer was added to 150 µL of whole blood into each well of the 96-deep-well plate. Samples were then mixed with multi-channel and incubated at RT for 20 minutes. An amount of 600 µL of PBS was then added to all wells and samples were centrifuged at 600 × *g* for 5 minutes. This step was repeated twice, then pellets were resuspended in 200 µL of PBS and transferred to a 96-well V-bottom plate for cell staining.

To process serum samples for ELISA, serum separation tubes were spun at 1,500 × *g* for 5 minutes to separate the serum from the clotted blood. Serum was then collected from the tubes and placed into a 96-well round-bottom plate. The serum was stored at −80°C until antibody ELISAs were performed.

### Tissue dissection and dissociation

After 14 and 40 days of immunization, spleen, inguinal, axillary, and brachial draining lymph nodes were collected from the side of the injection of each mouse and placed in PBS. The dLN was then transported on ice to Corner Therapeutics labs. The dLN was then processed using Miltenyi’s spleen dissociation kit according to the manufacturer’s protocol. In brief, the dLN from each mouse was transferred into the gentleMACS C Tube containing the enzyme mix. The dLN was then dissociated using the gentleMACS Program: program 37C_m_SDK_1. Cell suspensions were then collected and filtered through a 30-µM pre-separation filter. Single-cell suspensions were pelleted and treated with ACK buffer for 2 minutes at room temperature then filtered again through 30-µM pre-separation filters. Cells were counted using the Moxi automated counter. The dLN samples were then resuspended in complete RPMI (RPMI-1640 supplemented with 10% FBS, 100 U/mL penicillin-streptomycin, 2 mM L-glutamine, 1 mM sodium pyruvate, and 55 mM β-mercaptoethanol) at a density of 1.25E6 cells/mL.

Lungs were also collected after 40 days of the first immunization and were transported in PBS on ice to Corner Therapeutics labs. The lungs were stored in the tissue storage solution (Miltenyi Biotec) overnight. The next day, the lungs were processed using a lung dissociation kit (Miltenyi Biotec) as per the manufacturer’s protocol. In brief, the lobes of the lungs were separated and transferred to gentleMACS C tubes containing dissociation enzymes. The lungs were dissociated using the 37_m_LDK1 program on gentleMACS Dissociator. Cell suspensions were then collected and filtered through a 70-µM pre-separation filter. Cells collected from the lungs were counted using the Moxi automated counter. CD45^+^ cells were enriched from the lungs using anti-mouse CD45 beads (Miltenyi), then pooled CD45^+^ cells from 5 mice were resuspended in complete RPMI at a density of 1.25E6 cells/mL.

### OVA-specific antibody assessment

OVA-specific antibodies in the serum of mice receiving OVA-LNP immunization were assessed 7 days post-boost. OVA-specific total IgG, IgG1, and IgG2b were assessed using ELISA. Briefly, ELISA plates were coated with 10 µg/mL Endofit Ovalbumin (Invivogen) overnight, then washed and blocked with 2% bovine serum albumin. Plates were washed again, and then serum was added to the plates at a 1:500 dilution, followed by 1:5 dilutions completed for a total of 7 serum dilutions tested. Samples were washed and then incubated with detection antibody specific for IgG, IgG1, or IgG2b conjugated to HRP (Southern Biotech), to detect total, Th2, or Th1 skewing OVA-specific antibodies, respectively. Plates were washed, then incubated with TMB, and stop solution was added once color development was completed.

### Enzyme-linked immunosorbent spots assay

IFNγ ELISPOT plates (R&D Systems) were blocked with 200 µL/well complete RPMI for 45 minutes. At the end of blocking, media was discarded and 100 µL/well of either media alone or media containing 1 mg/mL of Ovalbumin PepTivator (Miltenyi Biotec) was added. Ovalbumin Peptivator is a pool of OVA peptides consisting mainly of 15-mer sequences with 11 amino acids overlap, covering the complete sequence of OVA. The dLN cell suspension was seeded at 5E5 cells/well in 100 µL and plates were incubated at 37°C for 18 hours. After incubation, cells were discarded and ELISPOTs were developed according to the manufacturer’s instructions. As described briefly, plates were washed four times with 1× wash buffer (R&D Systems) followed by a 2-hour incubation at room temperature with 100 µL/well of diluted detection antibody. Plates were washed four times and 100 µL/well of diluted streptavidin-alkaline phosphatase was added for 2 hours at room temperature. Plates were washed four times and 100 µL/well 5-bromo-4-chloro-3′ indolylphosphate p-toluidine salt and Nitro Blue Tetrazolium Chloride (BCIP/NBT) substrate was added. Plates were incubated for 1 hour at room temperature while being hidden from light. The substrate was removed and the plates were washed with deionized water. Plates were gently dried with Kimwipes and left to dry overnight at room temperature. The next day, plates were read on the S6 Universal M2 ELISPOT plate analyzer.

### IFNγ immunoassay

Single-cell suspension from the spleen and dLN of individual mice, or from pooled lungs of immunized mice were seeded into a round bottom 96-well plate in triplicates at a density of 1.25E5 cells/well. The cells were stimulated with either 20 µg/mL of Ovalbumin Peptivator or media alone for 96 hours. The culture supernatant was analyzed for IFNγ secretion using mouse IFNγ Lumit Kit (Promega) following the manufacturer’s protocol.

### Tetramer staining

3E5 cells from the dLN of each mouse, or 200 µL of processed blood samples (as described above), were plated into 96-well V-bottom plates. Cells were spun at 400 × *g* for 4 minutes and then washed with PBS at least once before cell staining. Cells were then resuspended in 100 µL PBS containing Live/Dead Aqua (1:1,000) and incubated for 20 minutes at 4°C. Cells were then washed and resuspended in 100 µL fluorescence activated cell sorting (FACS) buffer containing Fc block (1:100) for 10 minutes, then washed again with 100 µL FACS buffer. For tetramer staining, cells were resuspended in 100 µL of FACS buffer containing SIINFEKL-PE tetramer (1:20) and incubated at 37°C for 2 hours. For surface markers staining, cells were washed with 100 µL FACS buffer and spun for 4 minutes at 400 × *g*. The cells were stained with anti-mouse CD3 (1:200), anti-mouse CD4 (1:200), and anti-mouse CD8 (1:200) antibodies in FACS buffer (Table 2) for 20 minutes at 4°C. Cells were then washed and resuspended in 100 µL of 4% paraformaldehyde (PFA) to fix the cells for 20 minutes at room temperature. After fixation, cells are washed twice with FACS buffer and kept at 4°C overnight in 150 µL FACS buffer. Prior to sample acquisition on Symphony A3, CountBright Absolute Counting Beads (Invitrogen) are added to the samples to allow the measurement of the absolute number of cells.

## SUPPLEMENTAL MATERIAL

Supplemental Figures mbio.02506-23-s0001.pdf Figures S1–S5.

ASM does not own the copyrights to Supplemental Material that may be linked to, or accessed through, an article. The authors have granted ASM a non-exclusive, world-wide license to publish the Supplemental Material files. Please contact the corresponding author directly for reuse.
