# Supplementary material for: mRNAs encoding self-DNA reactive cGAS enhance the immunogenicity of lipid nanoparticle vaccines
Source: mBio. 2023 Nov 8;14(6):e02506-23. doi: 10.1128/mbio.02506-23 (PMC10746235; doi:10.1128/mbio.02506-23)
Supplement: Supplemental Figures — Figures S1–S5. [file mbio.02506-23-s0001.pdf]

Figure S1

Monocyte Purity Gating Strategy

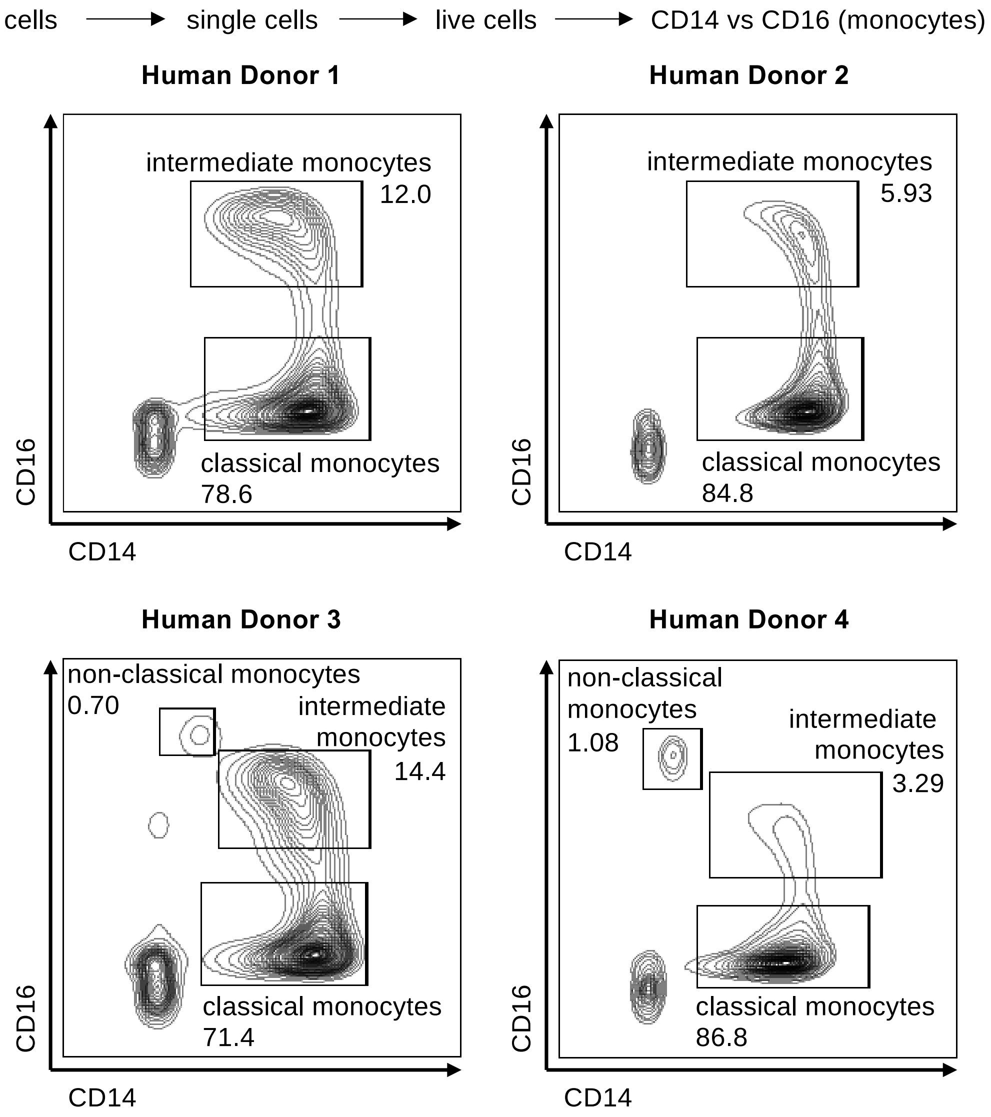

Figure S1. Purity of monocytes isolation from healthy leukopaks donors. Monocyte purity after isolation was assessed by flow cytometry. Flow plots represent the gating strategy used to determine the purity of monocytes by gating on $CD14^{+}CD16^{-}$ live cells. For all donors used in this paper, greater than 70% of the live cells isolated are classical monocytes, which can be differentiated into moDCs in the presence of GM-CSF and IL-4 recombinant cytokines.

Figure S2

MoDC Generation QC Gating Strategy: 6 days post monocyte differentiation

cells ⟶ single cells ⟶ live cells ⟶ CD11c vs CD209 (moDCs)

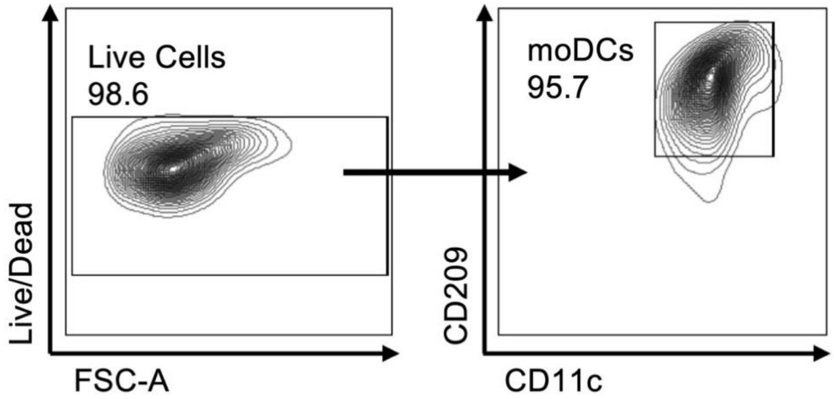

Figure S2. Efficiency of moDC differentiation 6 days post culture. Monocytes were differentiated for 6 days in the presence of GM-CSF and IL-4 to generate moDCs. moDC purity was assessed using flow cytometry. Flow plots represent the gating strategy used to determine the purity of moDC by gating on $CD209^{+}CD11c^{+}$ live cells. For all donors tested, moDC purity after 6 days was greater than 90%. These plots are representative of 4 donors.

Figure S3

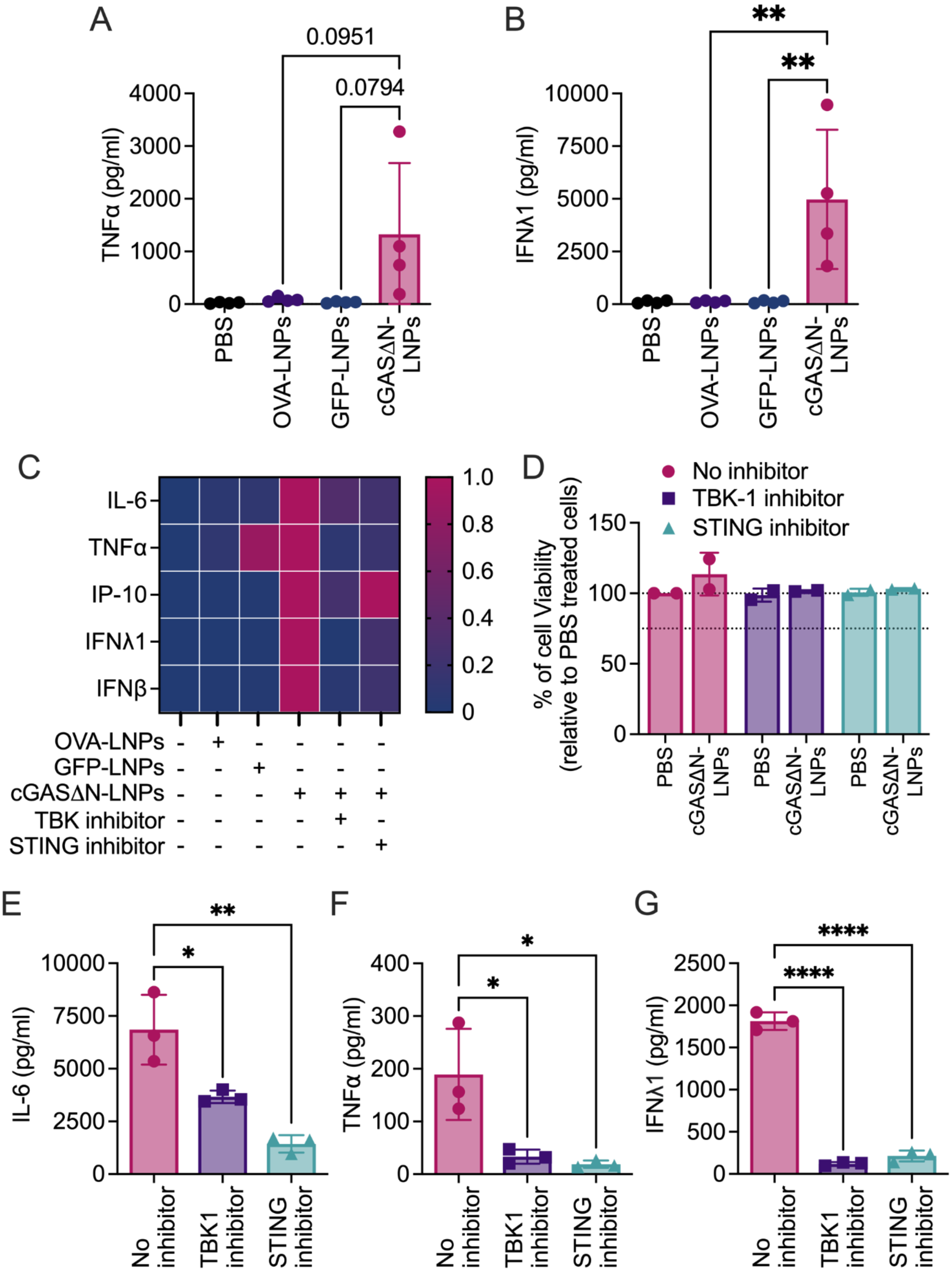
A
0.0951
0.0794
TNFα (pg/ml)
4000
3000
2000
1000
0
PBS
OVA-LNPs
GFP-LNPs
cGASΔN-LNPs
B
**
**
IFNλ1 (pg/ml)
10000
7500
5000
2500
0
PBS
OVA-LNPs
GFP-LNPs
cGASΔN-LNPs
C
IL-6
TNFα
IP-10
IFNλ1
IFNβ
1.0
0.8
0.6
0.4
0.2
0
OVA-LNPs - + - - - -
GFP-LNPs - - + - - -
cGASΔN-LNPs - - - + + +
TBK inhibitor - - - - + -
STING inhibitor - - - - - +
D
No inhibitor
TBK-1 inhibitor
STING inhibitor
% of cell Viability (relative to PBS treated cells)
150
100
50
0
PBS
cGASΔN-LNPs
PBS
cGASΔN-LNPs
PBS
cGASΔN-LNPs
E
**
*
IL-6 (pg/ml)
10000
7500
5000
2500
0
No inhibitor
TBK1 inhibitor
STING inhibitor
F
*
*
TNFα (pg/ml)
400
300
200
100
0
No inhibitor
TBK1 inhibitor
STING inhibitor
G
****
****
IFNλ1 (pg/ml)
2500
2000
1500
1000
500
0
No inhibitor
TBK1 inhibitor
STING inhibitor

Figure S3: cGASΔN-LNPs stimulate cGAS-STING pathway in human moDCs. Human moDCs were cultured in the presence of cGASΔN-LNPs (0.2μg/mL mRNA in well), GFP-LNPs (0.2μg/mL mRNA in well), or OVA-LNPs (1.0μg/mL mRNA in well). Cells were cultured for 24 hours after which cells and supernatants were collected to assess moDC activation. TNFα (A) and IFNλ1 (B) secretion were assessed using a multiplex cytokine bead array. Data points on graphs represent the average of a triplicate for each of four donors. Error bars show the standard deviation between donor cytokine secretion. MoDCs were cultured in the presence of cGASΔN-LNPs and STING pathway inhibitors MRT63707 (TBK1 inhibitor) and H-151 (STING inhibitor) for 24 hours, after which supernatants were collected to analyze cytokine secretion using a multiplex cytokine bead array. (C) Heat map of a second donor's response to cGASΔN-LNPs and STING pathway inhibitors. (D) Viability of human moDC donors after treatment with cGASΔN-LNPs and inhibitors. Viability is relative to PBS treated moDCs without inhibitor. IL-6 (E), TNFα (F) and IFNλ1 (G) secretion after STING inhibition. Data is representative of two donors. Comparisons between groups were completed using a one-way ANOVA with a Tukey post hoc test for multiple comparisons. *$p < 0.05$, **$p < 0.01$, and $p<0.0001$****.

Figure S4

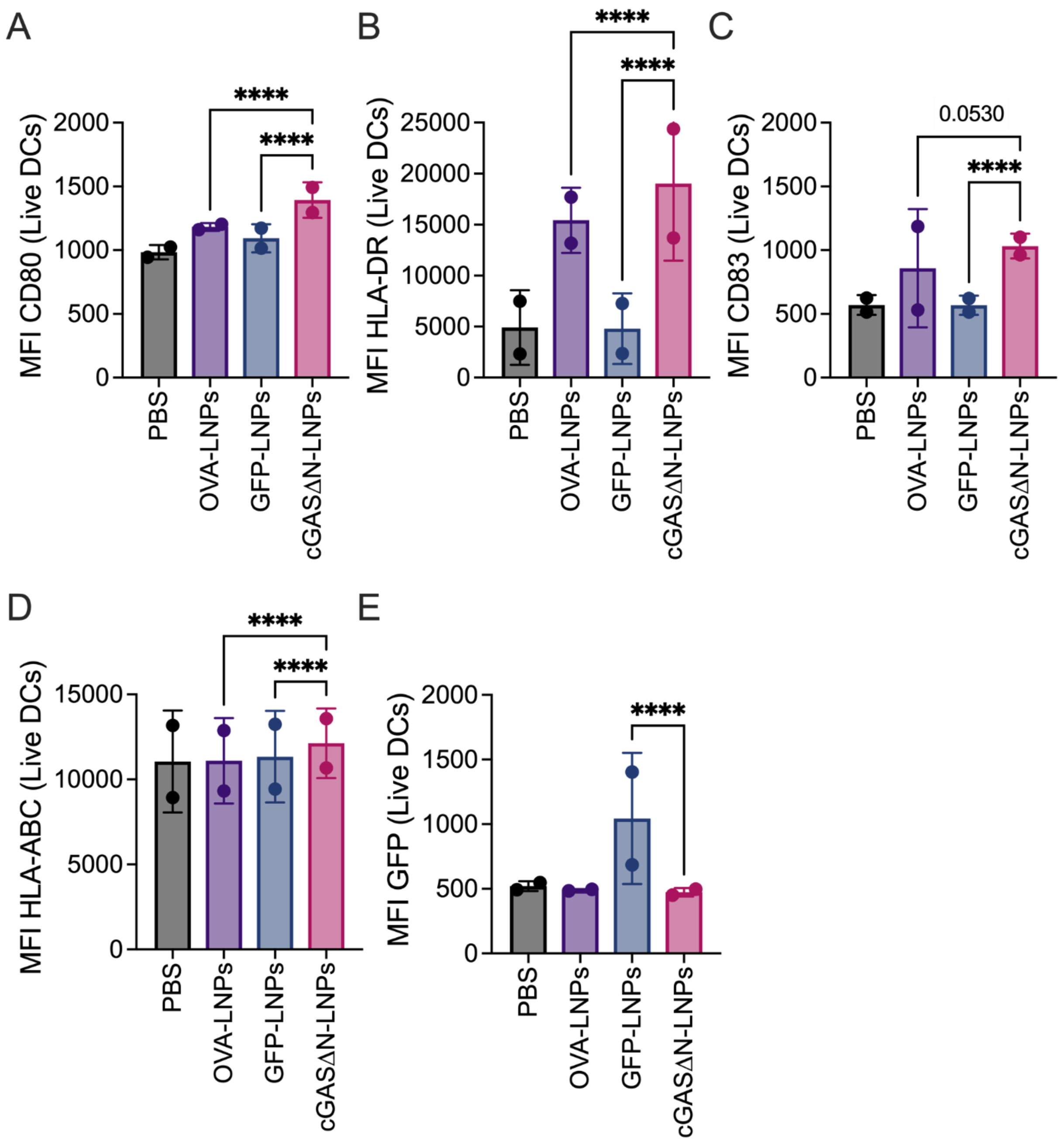

A
MFI CD80 (Live DCs)
2000
1500
1000
500
0
****
****
PBS
OVA-LNPs
GFP-LNPs
cGASΔN-LNPs
B
MFI HLA-DR (Live DCs)
25000
20000
15000
10000
5000
0
****
****
PBS
OVA-LNPs
GFP-LNPs
cGASΔN-LNPs
C
MFI CD83 (Live DCs)
2000
1500
1000
500
0
0.0530
****
PBS
OVA-LNPs
GFP-LNPs
cGASΔN-LNPs
D
MFI HLA-ABC (Live DCs)
15000
10000
5000
0
****
****
PBS
OVA-LNPs
GFP-LNPs
cGASΔN-LNPs
E
MFI GFP (Live DCs)
2000
1500
1000
500
0
****
PBS
OVA-LNPs
GFP-LNPs
cGASΔN-LNPs

Figure S4: cGAS∆N-LNPs induce moDC activation Human moDCs were cultured in the presence of cGAS∆N-LNPs (0.2μg/mL mRNA in well), GFP-LNPs (0.2μg/mL mRNA in well), or OVA-LNPs (1.0μg/mL mRNA in well). Cells were cultured for 24 hours after which cells and were collected to assess moDC activation markers. Flow cytometry was used to quantify surface marker expression on moDCs. MFI of CD80 (A), HLA-DR (B), CD83 (C), HLA-ABC (D), and GFP (E) expression was quantified by flow cytometry. Data points represent the MFI for each of two donors run in triplicate. Error bars show the standard deviation between donor surface marker expression. Comparisons between groups were completed using a one-way ANOVA with a Tukey post hoc test for multiple comparisons. P values are indicated between comparisons $p<0.0001$****

Figure S5

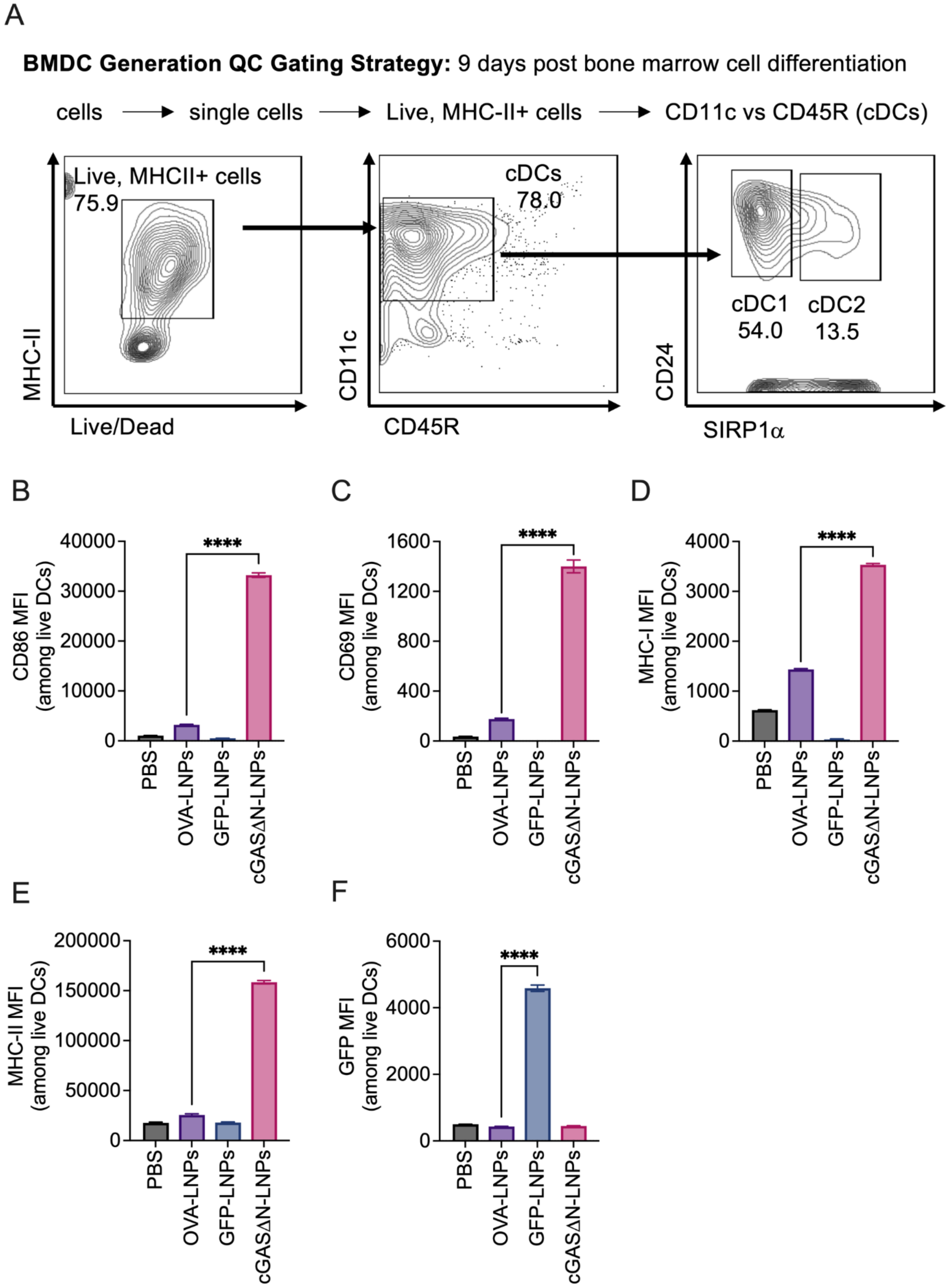
A
BMDC Generation QC Gating Strategy: 9 days post bone marrow cell differentiation
cells → single cells → Live, MHC-II+ cells → CD11c vs CD45R (cDCs)
Live, MHCII+ cells
75.9
MHC-II
Live/Dead
cDCs
78.0
CD11c
CD45R
cDC1
54.0
cDC2
13.5
CD24
SIRP1α
B
CD86 MFI (among live DCs)
C
CD69 MFI (among live DCs)
D
MHC-I MFI (among live DCs)
E
MHC-II MFI (among live DCs)
F
GFP MFI (among live DCs)
****
PBS
OVA-LNPs
GFP-LNPs
cGASΔN-LNPs

Figure S5. cGAS∆N-LNPs induce FLT3L-DC activation in vitro. (A) Bone marrow cells were differentiated for 9 days in the presence of FLT3L to generate cDCs. cDC purity was assessed using flow cytometry. Flow plots represent the gating strategy used to determine the purity of FLT3L-DCs by gating on $CD11c^{+}CD45R^{+}$ live MHC-II^{+} cells. The percentage of cDC1 and cDC2 among cDCs was assessed by flow cytometry. For all experiments represented in this paper, cDC purity after 9 days was greater than 78%, and the bulk population of cDCs was used for stimulation. (B-E) The median fluorescence intensity (MFI) of cells expressing (B) CD86 and (C) CD69, (D) MHC-I, (E) MHC-II, (F) GFP from triplicates are shown. Error bars represent the SEM for all DCs analyzed. Comparisons between groups were completed using a one-way ANOVA with a Tukey post hoc test for multiple comparisons. Data are representative of at least two experiments. ****$p < 0.0001$.
